# Circular dichroism for secondary structure determination of proteins with unfolded domains using a self-organising map algorithm SOMSpec†

**DOI:** 10.1039/d1ra02898g

**Published:** 2021-07-07

**Authors:** Adewale Olamoyesan, Dale Ang, Alison Rodger

**Affiliations:** Department of Molecular Sciences, Macquarie University NSW 2109 Australia dale.ang@mq.edu.au

## Abstract

Many proteins and peptides are increasingly being recognised to contain unfolded domains or populations that are key to their function, whether it is in ligand binding or material assembly. We report an approach to determine the secondary structure for proteins with suspected significant unfolded domains or populations using our neural network approach SOMSpec. We proceed by derandomizing spectra by removing fractions of random coil (RC) spectra prior to secondary structure fitting and then regenerating α-helical and β-sheet contents for the experimental proteins. Application to bovine serum albumin spectra as a function of temperature proved to be straightforward, whereas lysozyme and insulin have hidden challenges. The importance of being able to interrogate the SOMSpec output to understand the best matching units used in the predictions is illustrated with lysozyme and insulin whose partially melted proteins proved to have significant β_II_ content and their CD spectrum looks the same as that for a random coil.

## Abstract 2

Circular dichroism secondary structure fitting by analysing derandomized spectra using the SOMSpec approach then regenerating data for the original spectrum.

## Graphical abstract

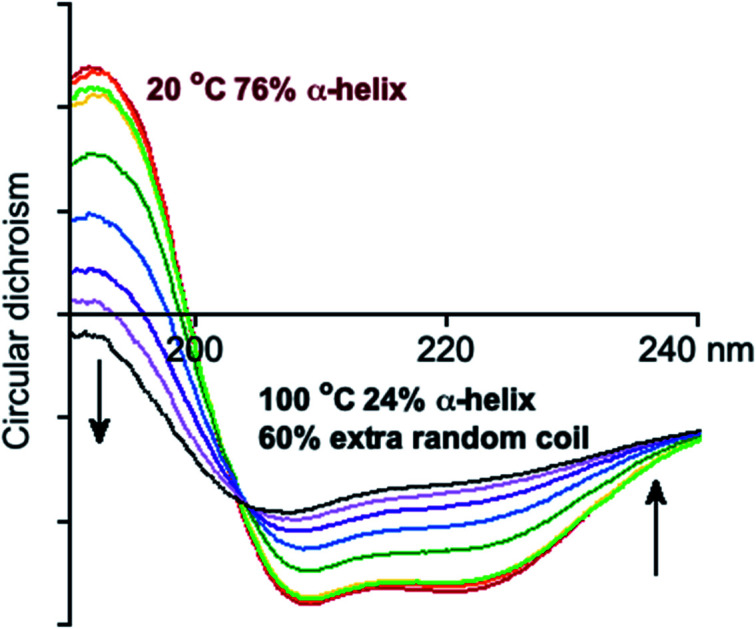

## Introduction

Proteins are essential molecules of life and play vital physiological roles in all living organisms. It is now an accepted fact that the function of a protein is dependent on its structure. However, about 50% of all the human proteins are postulated to contain unordered structure.^1,2^ Intrinsically disordered structures (IDPs) play crucial roles in signalling and molecular interactions,^3,4^ regulation of numerous pathways,^5–8^ cell and protein protection,^9–11^ and cellular homeostasis.^12,13^ IDPs also play a role in the ordered assembly of macromolecular machines such as the ribosome, in organization of chromatin, in assembly and disassembly of microfilaments and microtubules, in transport through the nuclear pore, in binding and transport of small molecules, in the functioning of protein and RNA chaperones, as flexible “entropic” linkers that separate functional protein domains and on the pathway from monomeric to assembled fibrils and other structures.^14–16^ Since the discovery of the essential cellular functions of intrinsically disordered proteins or regions, there have been considerable efforts to characterize and quantify unordered structure in proteins.^17^ The structure or potential structure of a protein is a key to its ability to be designed into new materials for biological applications. However, we still lack tools for analysing solution structures of proteins.

The most successful approaches to identifying secondary structure content from a spectrum include SELCON,^18^ CONTIN,^19^ and our own neural network approach originally called SSNN (secondary structure neural network) then generalised to SOMSpec (self-organising map for spectroscopy).^20–22^ The approaches all use a reference set of spectra for proteins with known solution phase structure (usually assumed to be that of the crystal structure).

The presence of unfolded structure is apparent in a CD spectrum to the experienced eye by the shift of a negative maximum from 208 nm (α-helix) or ∼217 nm (β-sheet) towards 200 nm and a smaller than usual 195 nm positive intensity. However, it should be noted that unfolded structures are spectroscopically indistinguishable from both polyproline II and β_II_ structures. We were disappointed when SOMSpec with reference sets such as Dichroweb's reference set 7 or SP175 reference set^23^ augmented by spectra for unfolded structures failed to provide satisfactory predictions for a well-folded protein to which unfolded peptides had been covalently linked.^24^ We hypothesised that the reason was because the folded protein and unfolded protein are on different parts of the map in the case of SOMSpec. SELCON also performed poorly, presumably due to not selecting unfolded reference spectra for proteins where the number of unfolded residues is a small percentage of the total. However, after subtracting appropriate fractions of an unfolded protein spectrum from the experimental one to produce a ‘derandomized’ spectrum, we were able to predict the structure of that artificial truncated protein and then regenerate a structure prediction for the original protein by reintroducing the random component. Among other results, this approach told us (not surprisingly) that the conjugation of a random peptide to the N-terminus (with a slight sequence change) unfolded some terminal residues of the parent protein.^24^

In this work, we have turned our previous *ad hoc* approach^24^ into a more systematic one that proceeds by removing variable amounts of a random coil spectrum from a protein CD spectrum until a good SOMSpec fit is obtained for the derandomised core protein. The same approach works for a population of proteins or peptides where some are folded and some unfolded. Then, the random coil component is added back in to determine the percentages of secondary structure motifs in the original protein/peptide. We apply the approach to analyse the secondary structures of proteins during melting curves, where it is known that random coil structures gradually appear. Application to proteins with natively unfolded domains is immediate. In addition, it provides a means of assessing whether proteins from different production approaches, for example, are fully folded or not. We have continued to work with SOMSpec rather then *e.g.* SELCON since, although the equality of the fits are similar,^20^ with SOMSPec it is a simple matter to identify which spectra are used in the fitting.

## Materials and methods

### Materials

Bovine serum albumin (BSA) and lysozyme were purchased from Sigma-Aldrich (Poole, UK). All solutions for the experiments were prepared using deionized water.

### Methods

Data were collected on approximately 0.1 mg mL^−1^ protein solutions in water and Δ*ε* for the spectra was determined using accurate literature Δ*ε*_222 nm_ values for 20 °C spectra. Samples were placed in 1 mm stoppered cuvettes and data collected with a Jasco J-1500 spectropolarimeter with a Peltier thermostatting unit (Hachioji, Japan) with temperature monitored on the cell block. The melting was monitored at 222 nm and wavelength spectra collected every 10 °C from 20–100 °C degrees at a ramp rate of 0.3 °C min^−1^ (which is sufficient to avoid hysteresis).

SOMSpec is a self-organising map approach to CD structure fitting that has been described in detail elsewhere.^20–22^ It essentially involves moving spectra into a reference set into locations of similar spectra shape on a 2-dimensional map than then placing an unknown in the best place on the map. The secondary structure of the map is determined to be that of its best matching node which in turn is derived as a weighted sum of the secondary structures of the nearest neighbour reference spectra on the map.

Input data for SOMSpec (the code is written in MatLab and available on request) was prepared by placing the Δ*ε* per molar residue CD data into a spreadsheet. To facilitate plotting of experimental and reference set data, the experimental spectra were truncated to a wavelength range of 240–190 nm with a step size of 1 nm (so 51 data points per spectrum). Input for SOMSpec requires a txt file that is in comma separated variable (csv) format with each spectrum placed in a column, which is then converted to txt format. The wavelength ranges (though not necessarily the data step size) of the reference set spectra and the test sets must match. In addition, each reference set member has its secondary structure annotations appended to its column in a consistent order (*e.g.* helix, sheet, turn *etc*.). In this work we used the SP175 reference set with data extracted from the Protein CD Data Bank (annotated with 5 structures: α-helix, β-sheet, bonded turn, bend and loop).^25^ SP175 was augmented by 4 unfolded and 2 fully helical structures as done in ref. 21 and 22. The best unfolded spectrum was derived from that of the KK peptide of ref. 26. We recently showed that, although one can annotate a protein with many independent structures, there is only enough information in a far UV circular dichroism (CD) spectrum to identify 3 types of structure, broadly α-helix or β-sheet or ‘other’.^27^ So we present results in terms only of α-helix, β-sheet, and other structures.

The SP175 proteins were put into columns in csv format with their structures forming 5 extra entries in each column. The files are then renamed as txt. SOMSpec was used to train the reference set with a map size of (50 × 50), 5 best matching units (BMUs), and 5 structures.^20–22^ We created a suite of MATLab modules to create the required baseline corrected derandomized input test files for SOMSpec with systematically varied amounts of the unfolded peptide spectrum subtracted as described in the ESI† and to post-process the output for the current work. The format of the output (*e.g*. Fig. 2 and 3) is a 2 dimensional representation of the Self Organising Map (SOM), in this case a 50 × 50 grid, where the positions of the reference proteins are indicated. The numbers on the SOMSpec output refer to the reference set proteins in the order in which the spectra appear in our input file (see ESI†). The 5 red dots are the nodes on the SOM that are the best matches to the test spectrum.

## Results and discussion

**Fig. 1:**
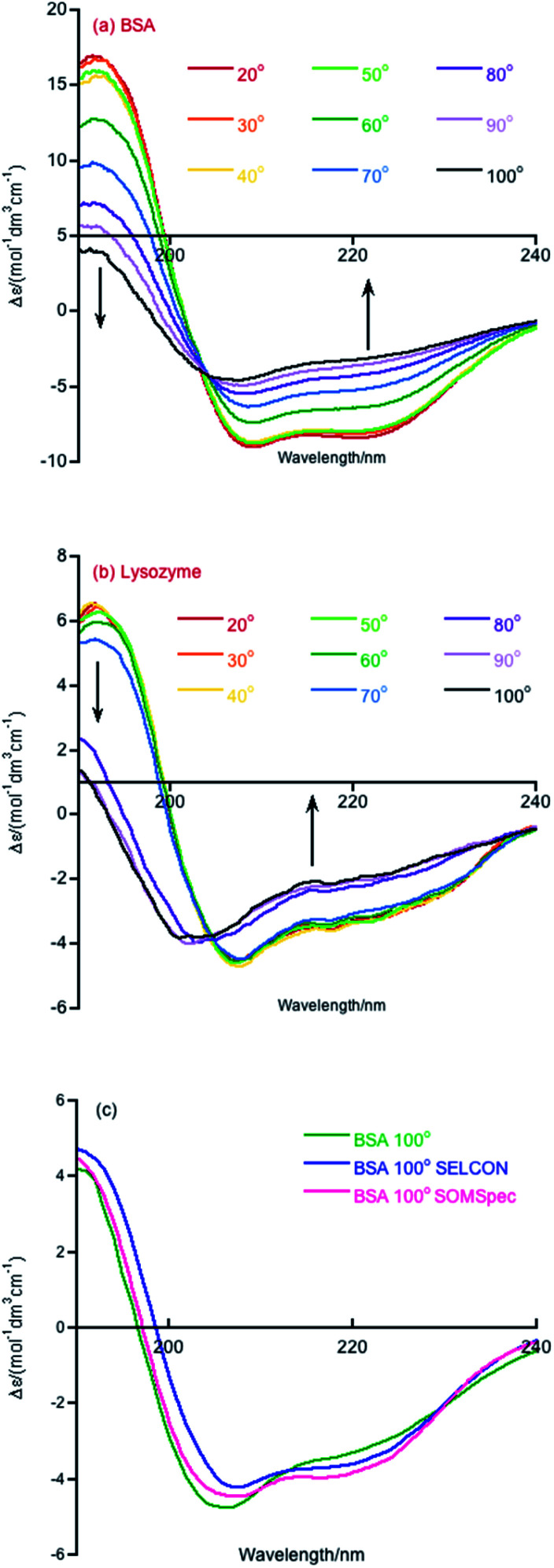
CD spectra of (a) BSA and (b) lysozyme over the temperature range of 20 to 100 °C in 10 °C steps. Data were collected on 0.1 mg mL^−1^ samples in water. (c) BSA 100° experimental spectrum overlaid with the SELCON and SOMSpec best predicted spectra.

Melting curve CD data are shown in Fig. 1a and b for BSA and lysozyme. BSA shows a gradual change above 50 °C whereas lysozyme shows a sharp transition between 70 °C and 80 °C. In both cases, the negative maximum moves towards 200 nm and the 190 nm intensity decreases as temperature increases, so it is likely that the change is due to gradual unfolding of parts of the protein. Directly applying SOMSpec and also SELCON with the augmented SP175 reference set gave an unsatisfactory overlay of experimental and predicted spectra as shown for 100 °C BSA (Fig. 1c). SOMSpec predicted 26% α-helix, 21% β-sheet and SELCON (*via* Dichroweb^23^) predicted 34% α-helix and 14% β-sheet, both with high spectral NRMSDs of respectively 0.05 and 0.25.

**Fig. 2:**
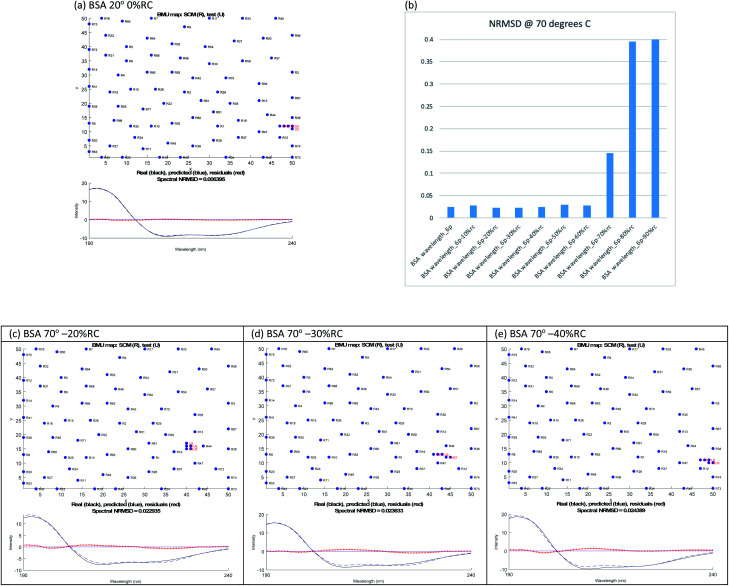
SOMSpec output for BSA (a) at 20 °C with 0% RC. (b) SOMSpec spectral NRMSDs for 70 °C BSA with different degrees of randomisation and output for 70 °C with (c) −20% RC, (d) −30% RC, and (e) −40% RC. The *x* and *y* axis labels indicate the self-organising map size and node positions for each run.

We therefore systematically derandomized the spectra by subtracting fractions of a random coil (RC) spectrum (10–90% in steps of 10%). The derandomized molar residue Δ*ε* was determined using1
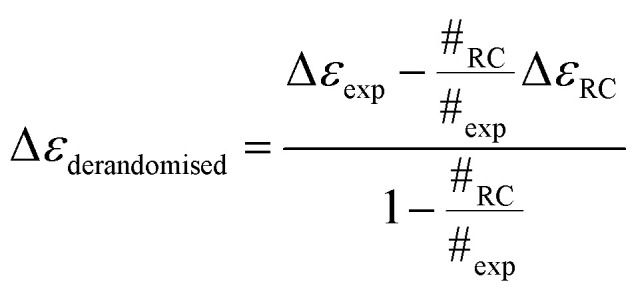
where Δ*ε*_RC_ is the CD spectrum of the random coil SufI-KK peptide MSLSKKQFIQASGIALCAGAVPLKASA,^26^ # denotes number of residues, exp denotes the full experimental protein on which data were collected. As outlined in the ESI,† this generated 90 spectra which we fitted with SOMSpec to generate 90 best predictions with NRMSDs and associate structure estimates. We discounted all fits with an NRMSD > 0.03 and then visually inspected low NRMSD structures for each temperature. The NRMSD plots are given in Fig. S4–S6 of the ESI.† For BSA most of the best choices are obvious (see *e.g.*Fig. 2(a)) and are summarised in Table 1.

**Table 1:** Spectral NRMSDs and structure predictions from BSA CD spectra as a function of temperature derived using derandomized spectra and structure predictions for the original spectra (where best fit requires >0% RC to be added). Column 2 indicates the NRMSD of the identified best fits. Columns 3 indicates the RC percentage added with the next 3 columns indicating the secondary structure of that modified spectrum (note these columns are empty for 0% RC added). The final columns indicate the secondary content of the original protein (0% RC) when the RC coil content has been added back in

BSA (°C)	Best NRMSD	RC added	Derandomized			Regenerated original protein		
			α-Helix	β-Sheet	Other	α-Helix	β-Sheet	Other
20	0.009	0%				0.76	0.00	0.24
30	0.006	0%				0.76	0.00	0.24
40	0.016	0%				0.73	0.01	0.26
50	0.015	0%				0.75	0.00	0.25
60	0.017	20%	0.77	0.00	0.23	0.62	0.00	0.38
70	0.024	30%	0.68	0.01	0.31	0.48	0.01	0.52
80	0.022	50%	0.77	0.00	0.23	0.39	0.00	0.62
90	0.023	50%	0.66	0.02	0.32	0.33	0.01	0.66
100	0.026	60%	0.61	0.04	0.35	0.24	0.02	0.74

Overall, the helix content decreases above 50 °C, but, interestingly even at 100 °C (cell holder temperature), BSA retains 24% helix content. In our experience, this is the case for most real proteins (as opposed to peptides). Another point to note is that the NRMSDs increase with temperature, though remaining below our nominated acceptable value of 0.03, reflecting the increasing difficulty of SOMSpec to find a perfect place on the maps for the increasingly derandomized proteins (some of whose non-random content is of very low intensity so the derandomised spectra are very noisy).

The ‘right’ answer is not always immediately obvious. The NRMSDs for BSA 70° spectra, *e.g*., are similar for −20% RC, −30% RC and −40% RC (Fig. 2(b)). Placing emphasis on the shape between 210 nm and 190 nm (Fig. 2(c)–(e)), particularly considering where the spectrum is zero and how the positive and negative maxima overlay, we selected −30% RC as optimum even though its NRMSD is fractionally higher than −20% RC. A refinement with percentages between 20% and 40% could be implemented.

**Fig. 3:**
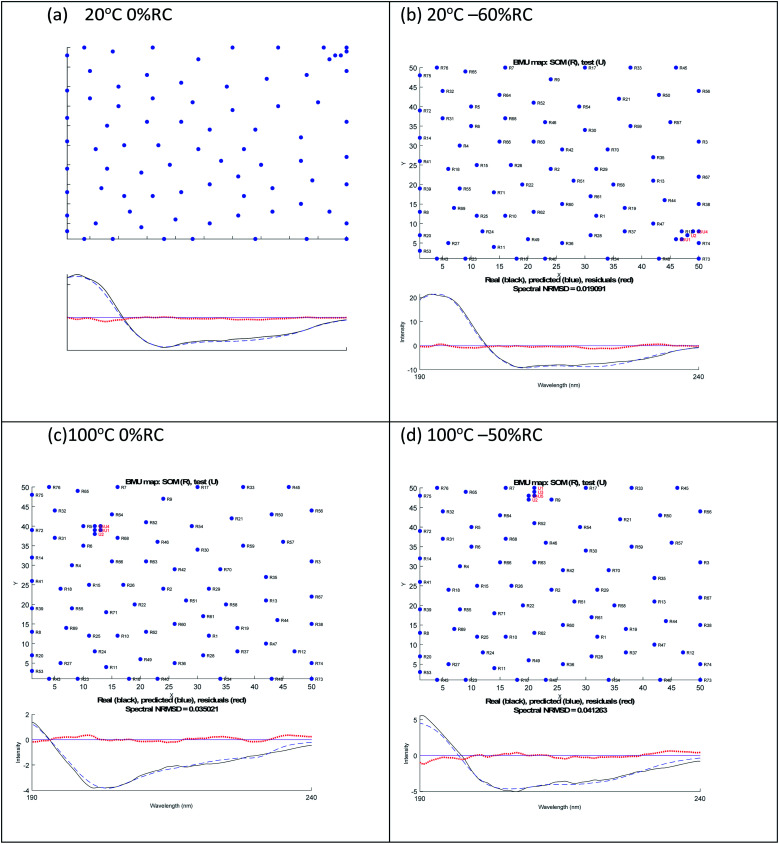
SOMSpec output for lysozyme at 20 °C (a) 0% RC, (b) −60% RC and at 100 °C (c) 0% RC, (d) −50% RC. The *x* and *y* axis labels indicate the self-organising map size.

A similar analysis for lysozyme proved to be more challenging than for BSA. For example, as summarised in Table 2, the 20 °C 0% RC result is 39% α-helix and 16% β-sheet which is close to the crystal structure of 40% and 10% respectively. However, the 20 °C −60% RC has a slightly lower NRMSD with (regenerated) 51% α-helix and 0% β-sheet. We prefer the 20 °C 0% RC fit because it is slightly better (Fig. 3(a) and (b)) with a less obvious 222 nm negative maximum following the atypical 222 nm region spectral shape of lysozyme.

**Table 2:** Spectral NRMSDs and structure predictions from lysozyme CD spectra as a function of temperature derived using the original data and derandomized spectra (where best fit requires >0% RC to be added). Column identity is as for Table 1. Bold indicates preferred values where more than one option gave a reasonable fit as discussed in the text

Lysozyme (°C)	Best NRMSD	RC added	Derandomized			Regenerated original protein		
			α-Helix	β-Sheet	Other	α-Helix	β-Sheet	Other
20	0.021 (0.019)	0%				**0.39**	**0.16**	**0.45**
		(60%)	(0.85)	(0)	(0.15)	0.34	0.00	0.66
30	0.021 (0.019)	0%				**0.39**	**0.16**	**0.45**
		(60%)	(0.85)	(0)	(0.15)	0.34	0.00	0.66
40	0.022 (0.019)	0%				**0.39**	**0.16**	**0.45**
		(60%)	(0.85)	(0)	(0.15)	0.34	0.00	0.66
50	0.023 (0.020)	0%				**0.39**	**0.16**	**0.45**
		(60%)	(0.85)	(0)	(0.15)	0.34	0.00	0.66
60	0.0234	0%				**0.39**	**0.16**	**0.45**
	(0.0237)	(60%)	(0.86)	(0)	(0.14)	(0.34)	(0)	(0.66)
70	0.023	10%	0.39	0.15	0.46	**0.35**	**0.14**	**0.51**
80	0.038	70%	0.64	0.02	0.34	0.19	0.00	0.80
	0.045	40%	0.27	0.20	0.52	**0.16**	**0.12**	**0.71**
90	0.036	0%				0.11	0.34	0.55
	0.044	(50%)	0.26	0.21	0.53	**0.13**	**0.11**	**0.76**
100	0.035	0%				0.11	0.34	0.55
	0.041	50%	0.26	0.21	0.53	**0.13**	**0.11**	**0.76**

By way of contrast when we consider the 100° data, we again have similar quality fits for 0% RC and −50% RC (Fig. 3(c) and (d) with the NRMSD values affected by noisy data) but here we prefer −50% RC. Both options indicate that by 100°, lysozyme has lost 30% of its helical content. However, at first sight the β-sheet content is very different. Based on the details of the SOMSpec output we prefer the −50% RC of 11% β-sheet: the BMUs for 100° 0% RC are mainly α-chymotrypsin and chymotrypsinogen, both of which Sreerama and Woody^28^ categorize as β_II_ proteins.

The CD-significance of β_II_ proteins is that the β-sheet content has a spectral form similar to that of unfolded proteins so unfolded and β_II_ cannot be distinguished. Thus any time a β_II_ protein is a BMU, we must ask whether the protein is β_II_, random coil or indeed polyproline II. This analysis provides an understanding of the relatively low (compared to typical proteins) 222 nm 20 °C signal of lysozyme, as its β_II_ structure shifts CD intensity from the 218 nm region to the 200 nm region. It should be noted that the above argument needs to be inverted to override the 80° −70% RC data in favour of 80° −40% RC (Table 2).

Based on CD structure fitting we previously speculated that insulin was beginning to form precursor amyloid fibre structures during a melting experiment.^29^ Given the above lysozyme results we repeated the analysis of insulin using SOMSPec and the augmented SP175 reference set used in this work (Dichroweb's reference set 7 was used previously)^29^ and examined the BMU proteins carefully. The new and old α-helix results without any RC added as a function of temperature are very similar. The β-sheet is similar until about 80° after which they are larger with SP175 – however, the NRMSDs are high (∼0.1). Not surprisingly the 20° fit is dominated by the insulin in the reference set, however, the closest proteins to *e.g*. the 110° spectrum were chymotrypsin, elastase, and ferrodoxin (proteins 5, 31, and 32 in the SOMSpec output maps *e.g.* top left of Fig. 2). α-Chymotrypsin and elastase are in the β_II_ list of ref. 28 and ferrodoxin looks like a very unfolded spectrum so its structural assignment based on a 100 K (ref. 30) crystal structure is misleading.

In contrast to lysozyme, when we attempted to derandomize insulin spectra by subtracting fractions of the unfolded KK spectrum, the lowest NRMSDs are still relatively high (see ESI†) which normally means that the structure predictions are only indicative. However, in this case, when we consider 2 or more of the lowest NRMSD predictions for each temperature (often with significantly differing percentages of randomisation), the α-helix predictions of the regenerated protein are within a few percent (see ESI†). For example, at 90° the predictions, *via* 20% and 30% derandomisation, are 20% and 18% α-helix and at 80°, *via* 10% and 60% derandomisation, are 22% and 16% α-helix. In these cases, the β-sheet predictions are 18% and 15% and the much more diverse 20% and 6% respectively. In the 80° case, different parts of the map are identified for the best matching units: near α-chymotrypsin for −10% RC *versus* near glycogen (which is 49% helical) for −60% RC. The resolution of this disparity is that 50% of α-chymotrypsin is β_II_ (not standard β) which is spectroscopically similar to 50% of RC added to glycogen. So, the two apparently different fits are indicative of the presence of either random coil or β_II_ or both – which CD cannot distinguish.

## Conclusions

Conformational changes in BSA, lysozyme and insulin during thermal denaturation in aqueous solution were examined by combination of computational analysis and CD spectroscopy. As visual inspection suggested an increase in random coil content of spectra, we proceeded by derandomizing the spectra by subtracting known fractions of a random coil spectrum prior to structure fitting using our algorithm SOMSpec. The fit for the original spectrum was then regenerated by adding the random coil back in. To assess the goodness of the spectral fit in instances where the NRMSDs are very similar, we complemented the fitting program with a visual inspection of the overlay of experimental data and model spectra. BSA was fairly straightforward to analyse. The lysozyme study indicates both the power and the pitfalls of the derandomisation/regeneration approach with the pitfalls being able to be mitigated by inspecting the SOMSpec output in detail and considering the nature of the best matching reference spectra. In one case, removing 0% RC and 50% RC were equally effective, with similar α-helix content for the original spectrum predicted but very different β-sheet. The resolution of this type of anomaly is that β_II_ has a spectral form similar to unfolded proteins so if β_II_ is present, β-sheet and random coils need to be considered together. Our previous speculations about the apparent increase of insulin β-sheet with increasing temperature proved to be of this kind.

It should be noted that the final absolute predictions of RC content are dependent on the intensity of random coil spectrum used being of appropriate magnitude, though the derandomisation and structure fitting is not impacted, so all results are internally consistent. The importance of inspection of the fitting maps to assess the roles of β_II_, polyproline and random coil must be emphasised. A combination of the self-organising map approach and human intervention provides an effective tool for analysing solution structures of proteins, particularly those being rearranged *via* random coil structures for new applications in material science.

## Author contributions

AO undertook all the calculations. DA designed and wrote the code. AR conceptualised the programme of work, analysed the outputs, and wrote the manuscript.

## Conflicts of interest

There are no conflicts to declare.

## Supplementary Material

RA-011-D1RA02898G-s001
