# Supplementary material for: Circular dichroism for secondary structure determination of proteins with unfolded domains using a self-organising map algorithm SOMSpec
Source: RSC Adv. 2021 Jul 7;11(39):23985–91. doi: 10.1039/d1ra02898g (PMC9036635; doi:10.1039/d1ra02898g)
Supplement: RA-011-D1RA02898G-s001 [file RA-011-D1RA02898G-s001.pdf]

SUPPLEMENTARY INFORMATION

Received 00th January 20xx,
Accepted 00th January 20xx

DOI: 10.1039/x0xx00000x

Circular dichroism for secondary structure determination of proteins with unfolded domains using a self-organising Map SOMSpec

Adewale Olamoyesan, Dale Ang, Alison Rodger

Electronic Supplementary Information

Table of Contents

1. SELCON output for BSA 100°C 0%RC 3

2. SOMSpec secondary structure prediction output for BSA 4

3. SOMSpec secondary structure prediction output for Lysozyme 7

4. SOMSpec secondary structure prediction output for insulin 10

5. SOMSpec NRMSD output for BSA 13

6. SOMSpec NRMSD output for lysozyme 16

7. SOMSpec NRMSD output for insulin 19

8. SOMSpec model and experimental spectra for BSA 22

9. SOMSpec model and experimental spectra for lysozyme 26

10. SOMSpec model and experimental spectra for insulin 30

11. Procedure for preparing input and summarising output for SOMSpec 34

CD/UV/IR: code for baseline correction of spectra. 41
Delta Epsilon: code for conversion of CD spectra in units mdeg to delta epsilon. 42
Scaling Factor: code for scaling of CD spectra using given factor(s) for concentration or conversion to delta epsilon. 44
Disordered: code for derandomization of spectra by removing varying fractions of random coil spectrum. 45
Concatenate: code for matching xlsx files together 46
Convert: code for the reading of txt files output of SOMSpec and output xlsx files 47

Please do not adjust margins

This journal is © The Royal Society of Chemistry 20xx

Please do not adjust margins

Please do not adjust margins

1. SELCON output for BSA 100°C 0%RC

Structure fitting output for BSA 100°C 0%RC (random coil) using SELCON 3 (The Self-Consistent Method) with Reference dataset: SP175 *via* the Dichroweb server [1] is given in Table S1.

Table S1. SELCON 3 secondary structure prediction for BSA at 100°C with 0%RC removed. NRMSD:0.245

Result	Helix1	Helix2	Strand1	Strand2	Turns	Unordered	Total
**Guess**	0.203	0.181	0.066	0.048	0.127	0.376	**1.001**
**SVD**	0.184	0.142	0.062	0.062	0.127	0.337	**0.914**
**Convergent**	0.163	0.146	0.105	0.075	0.142	0.371	**1.002**
**Stage2**	**0.163**	**0.146**	**0.105**	**0.075**	**0.143**	**0.371**	**1.003**
**final**	**0.183**	**0.164**	**0.079**	**0.062**	**0.159**	**0.365**	**1.012**

34% helix, 14% sheet; 52% other

This journal is © The Royal Society of Chemistry 20xx

Please do not adjust margins

2. SOMSpec secondary structure prediction output for BSA

The SOMSpec secondary structure predictions for BSA as a function of temperature for each percentage RC content subtracted are shown in Figures S1. Figure S1a is for the original protein, whereas Figures S1(b–j) are the regenerated proteins that are obtained by adding back the fraction of RC removed during derandomization.

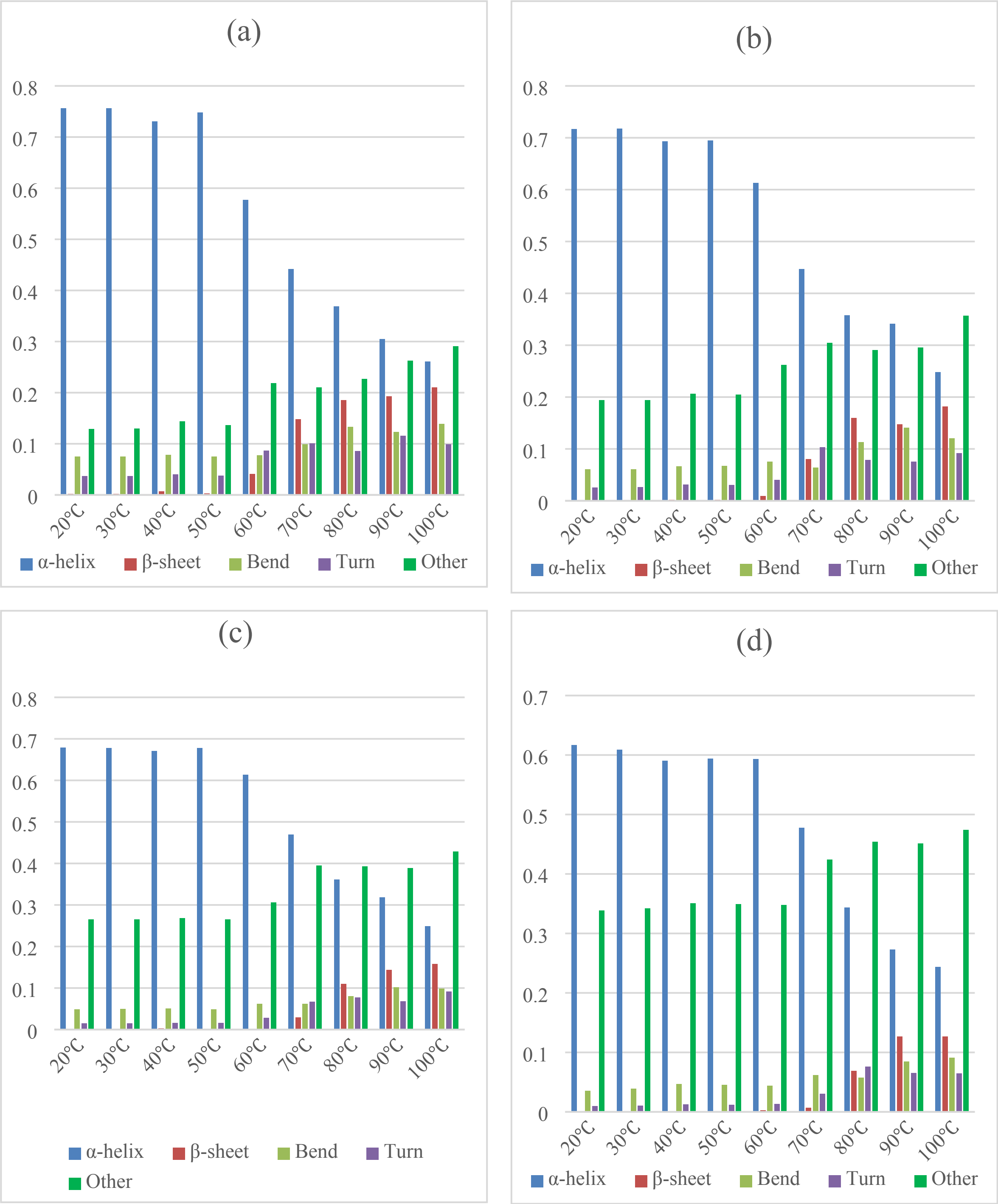

Please do not adjust margins

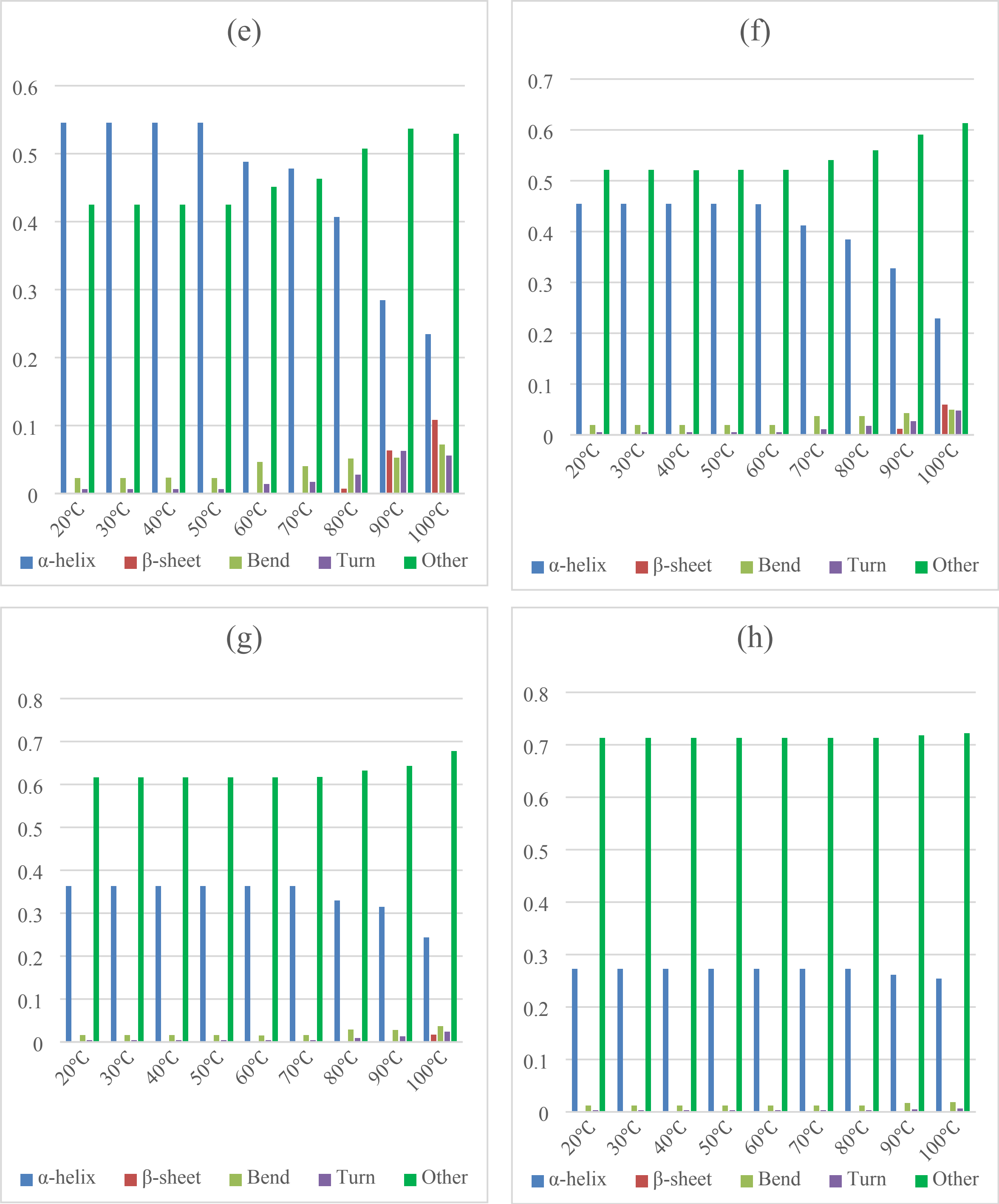

This journal is © The Royal Society of Chemistry 20xx

Please do not adjust margins

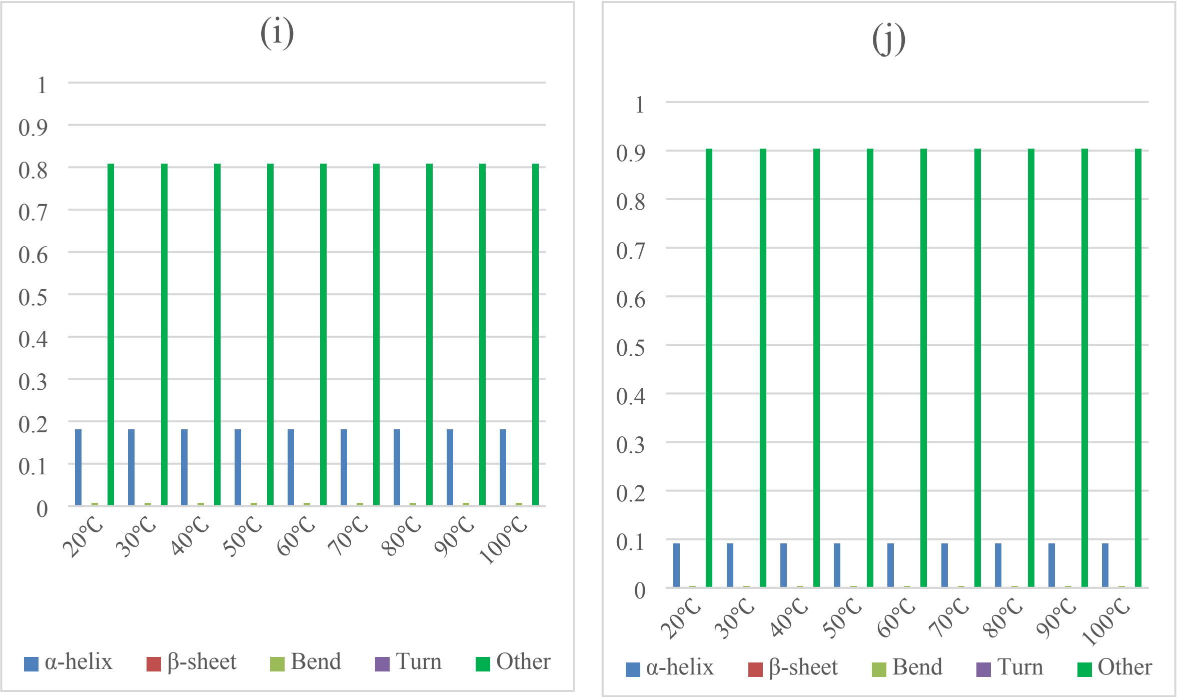

Figure S1. Secondary structure predictions for regenerated BSA as a function of temperature. The SOMSpec predictions for the derandomized BSA spectra were scaled to account for the removed RC percentage and then then RC percentage added to the Other category. (a) 0%, (b) 10%, (c) 20%, (d) 30%, (e) 40%, (f) 50%, (g) 60%, (h) 70%, (i) 80%, (j) 90% RC removed for the prediction process and added back to the derandomized BSA predictions.

3. SOMSpec secondary structure prediction output for Lysozyme

The SOMSpec secondary structure predictions for lysozyme as a function of temperature at each percentage RC content subtracted are shown in Figures S2. Figure S2a is for the original protein, whereas Figures S2(b-j) are the regenerated proteins that are obtained by adding back the fraction of RC removed during derandomization.

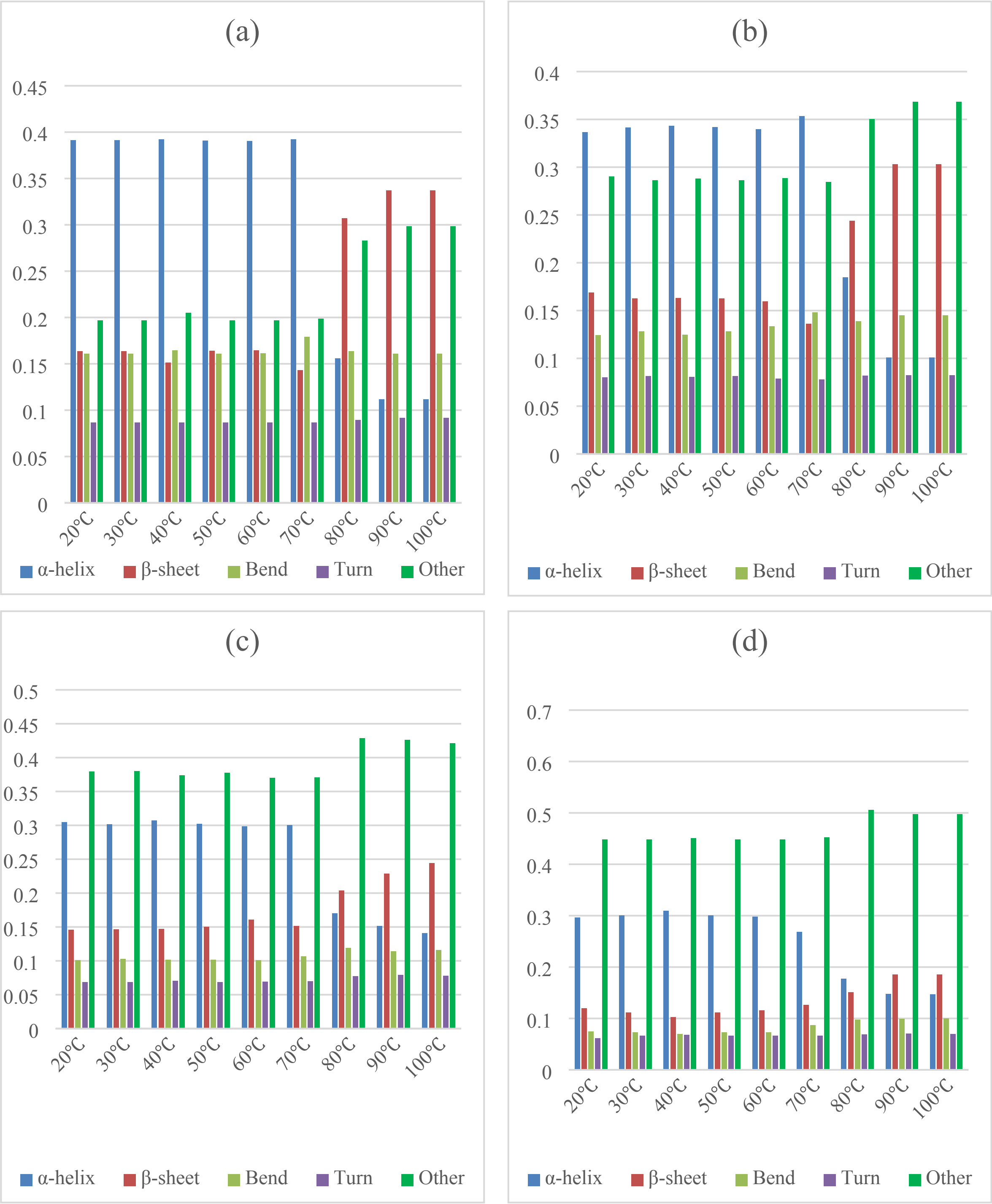

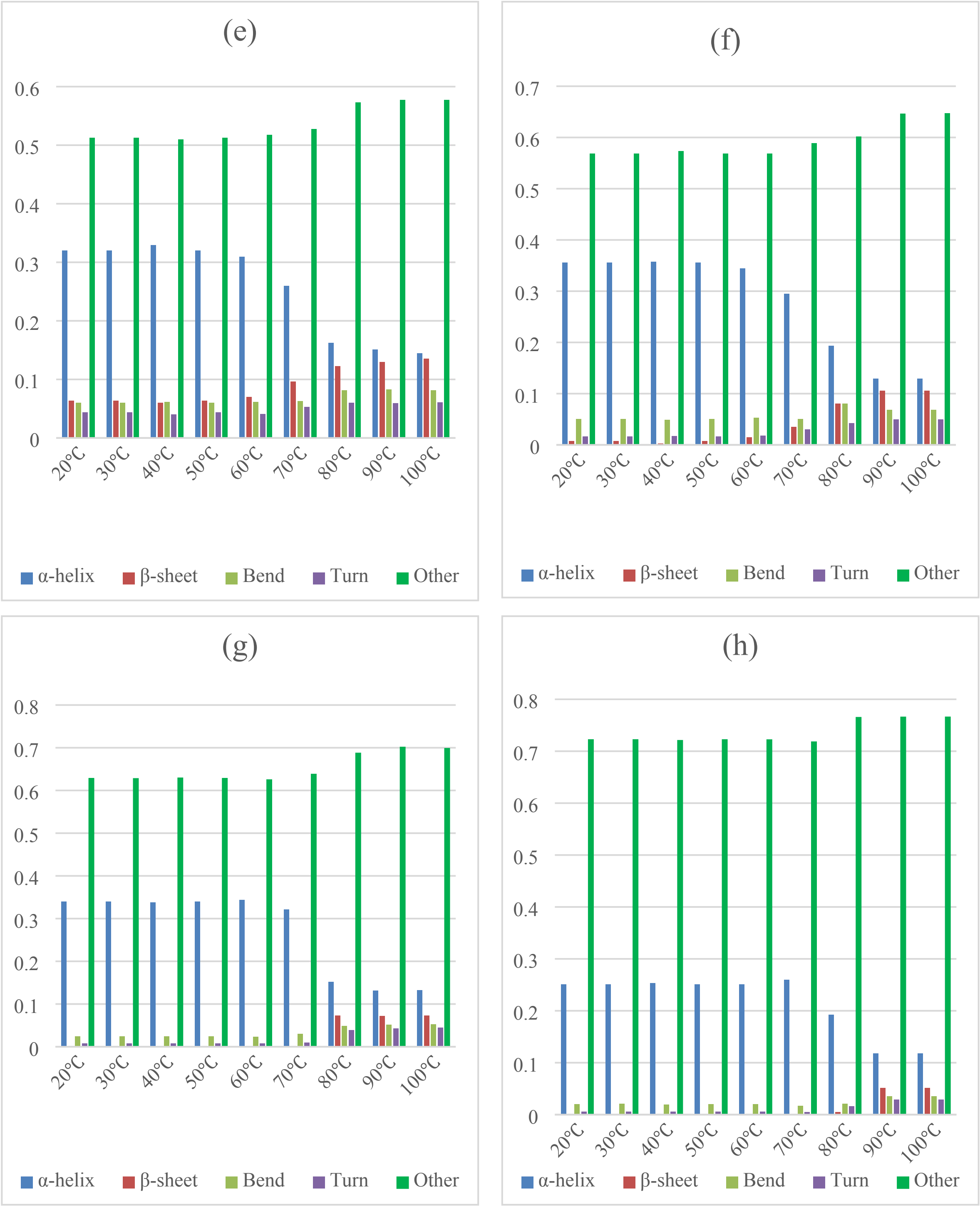

This journal is © The Royal Society of Chemistry 20xx

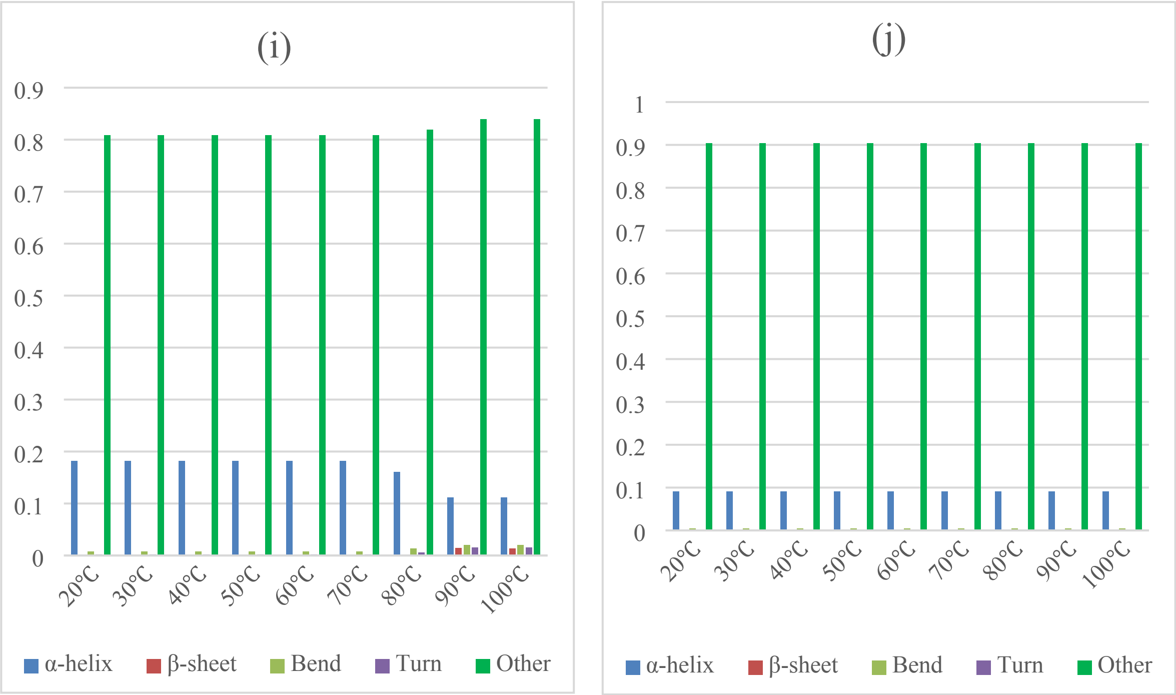

Figure S2. Secondary structure predictions for regenerated lysozyme as a function of temperature. The SOMSpec predictions for the derandomized lysozyme spectra were scaled to account for the removed RC percentage and then then RC percentage added to the Other category. (a) 0%, (b) 10%, (c) 20%, (d) 30%, (e) 40%, (f) 50%, (g) 60%, (h) 70%, (i) 80%, (j) 90% RC removed for the prediction process and added back to the derandomized lysozyme predictions.

4. SOMSpec secondary structure prediction output for insulin

The SOMSpec secondary structure predictions for insulin as a function of temperature at each percentage RC content subtracted are shown in Figures S3(a-. Figure S2a is for the original protein, whereas Figures S3(b-j) are the regenerated proteins that are obtained by adding back the fraction of RC removed during derandomization.

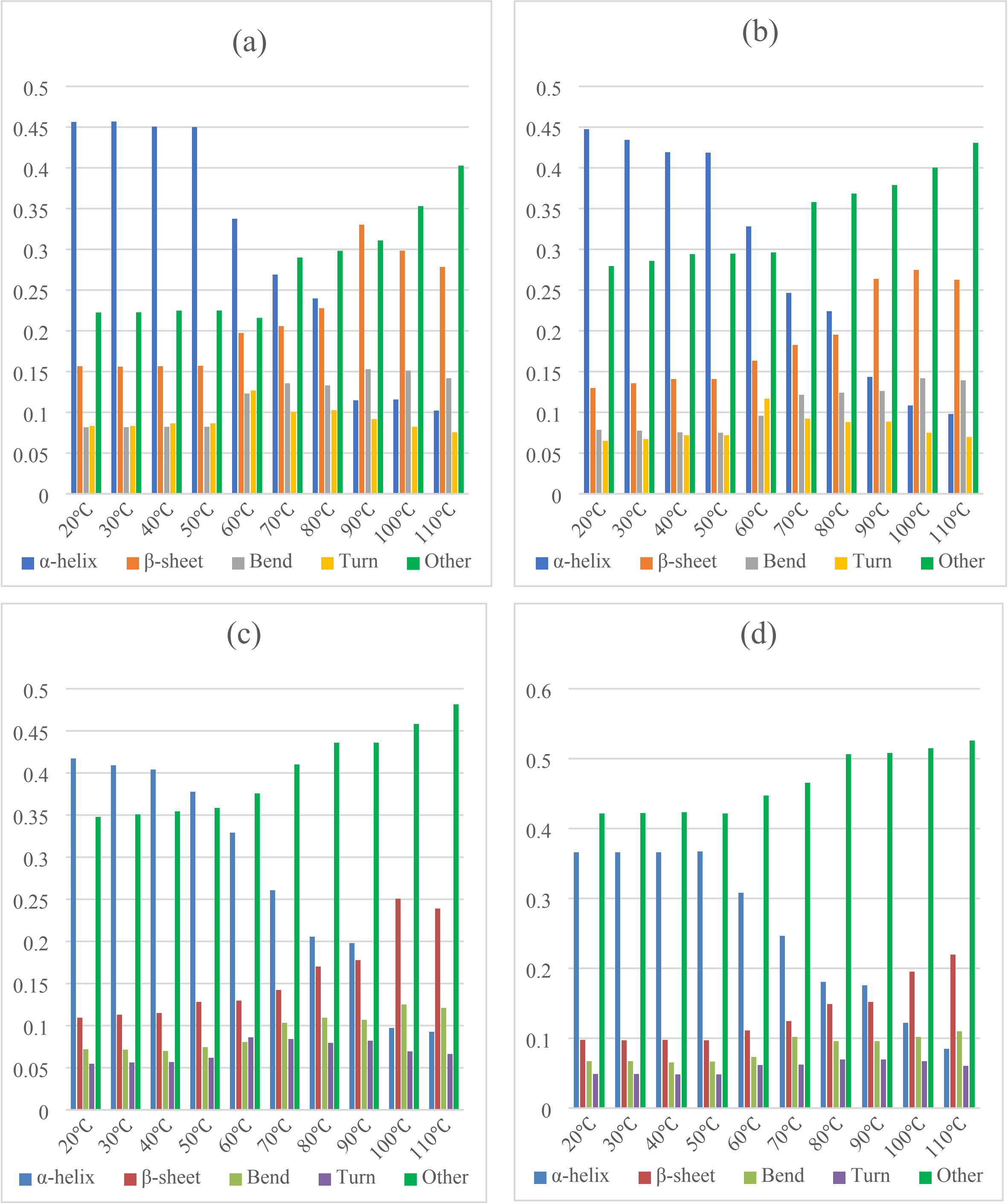

This journal is © The Royal Society of Chemistry 20xx

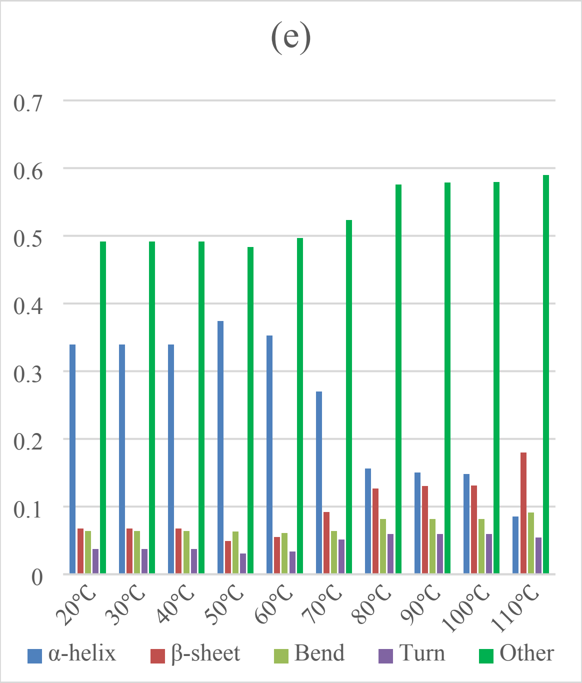
(e)
0.7
0.6
0.5
0.4
0.3
0.2
0.1
0
20°C
30°C
40°C
50°C
60°C
70°C
80°C
90°C
100°C
110°C
α-helix
β-sheet
Bend
Turn
Other

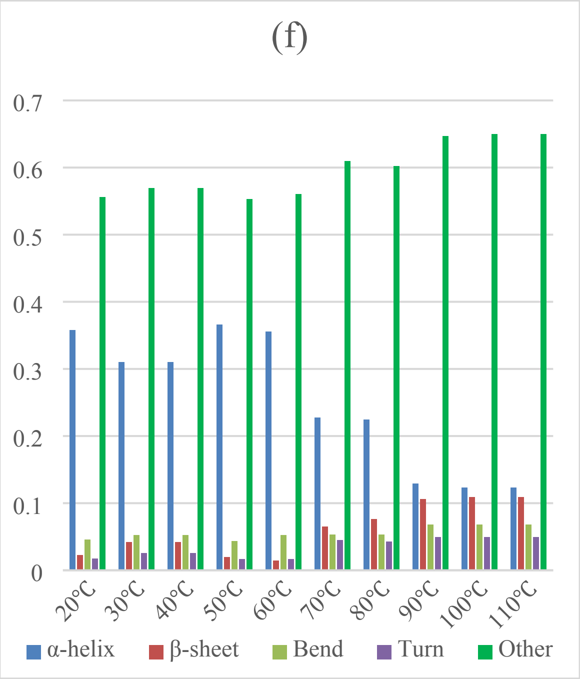
(f)
0.7
0.6
0.5
0.4
0.3
0.2
0.1
0
20°C
30°C
40°C
50°C
60°C
70°C
80°C
90°C
100°C
110°C
α-helix
β-sheet
Bend
Turn
Other

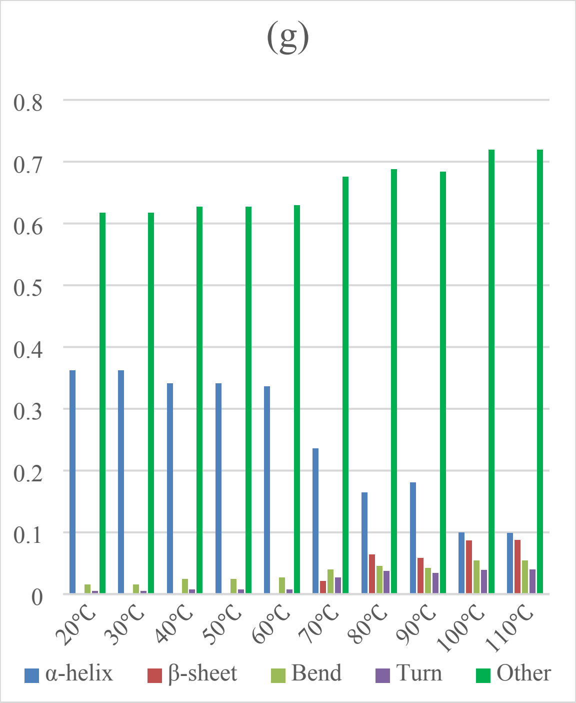
(g)
0.8
0.7
0.6
0.5
0.4
0.3
0.2
0.1
0
20°C
30°C
40°C
50°C
60°C
70°C
80°C
90°C
100°C
110°C
α-helix
β-sheet
Bend
Turn
Other

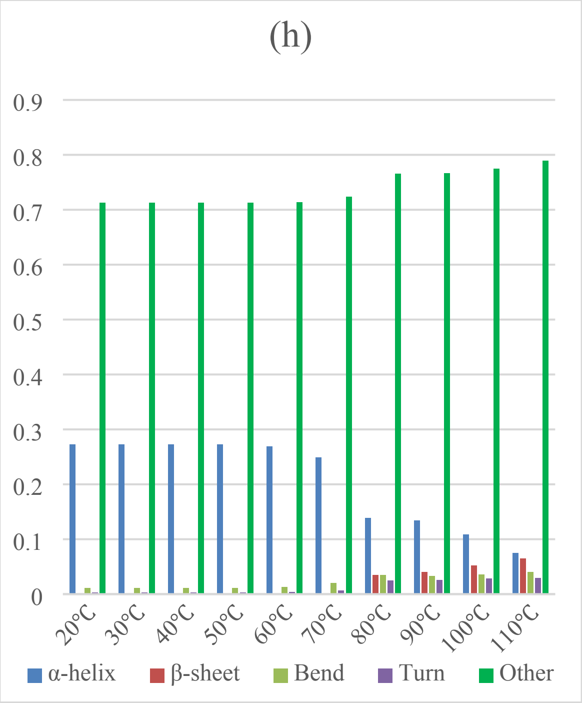
(h)
0.9
0.8
0.7
0.6
0.5
0.4
0.3
0.2
0.1
0
20°C
30°C
40°C
50°C
60°C
70°C
80°C
90°C
100°C
110°C
α-helix
β-sheet
Bend
Turn
Other

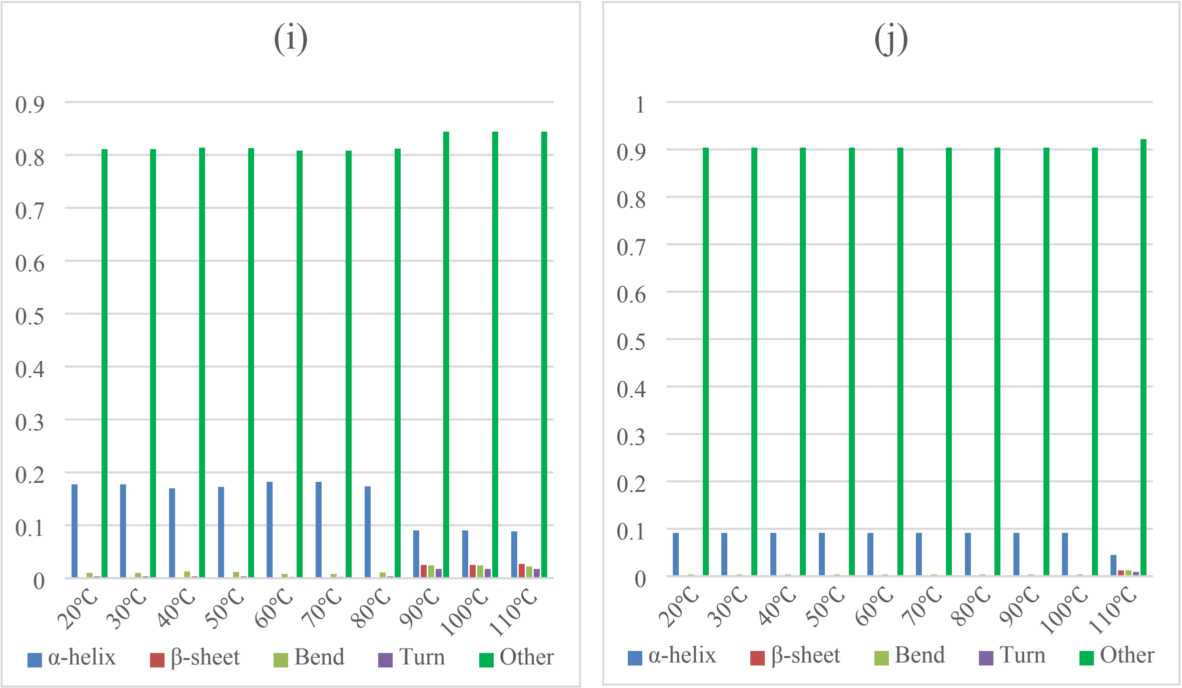

Figure S3. Secondary structure predictions for regenerated insulin as a function of temperature. The SOMSpec predictions for the derandomized insulin spectra were scaled to account for the removed RC percentage and then then RC percentage added to the Other category. (a) 0%, (b) 10%, (c) 20%, (d) 30%, (e) 40%, (f) 50%, (g) 60%, (h) 70%, (i) 80%, (j) 90% RC removed for the prediction process and added back to the derandomized lysozyme predictions.

This journal is © The Royal Society of Chemistry 20xx

Please do not adjust margins

5. SOMSpec NRMSD output for BSA

Figures S4 shows the spectral NRMSDs of the best model spectrum from the derandomised BSA experimental spectra with varying percentage RC removed at each temperature.

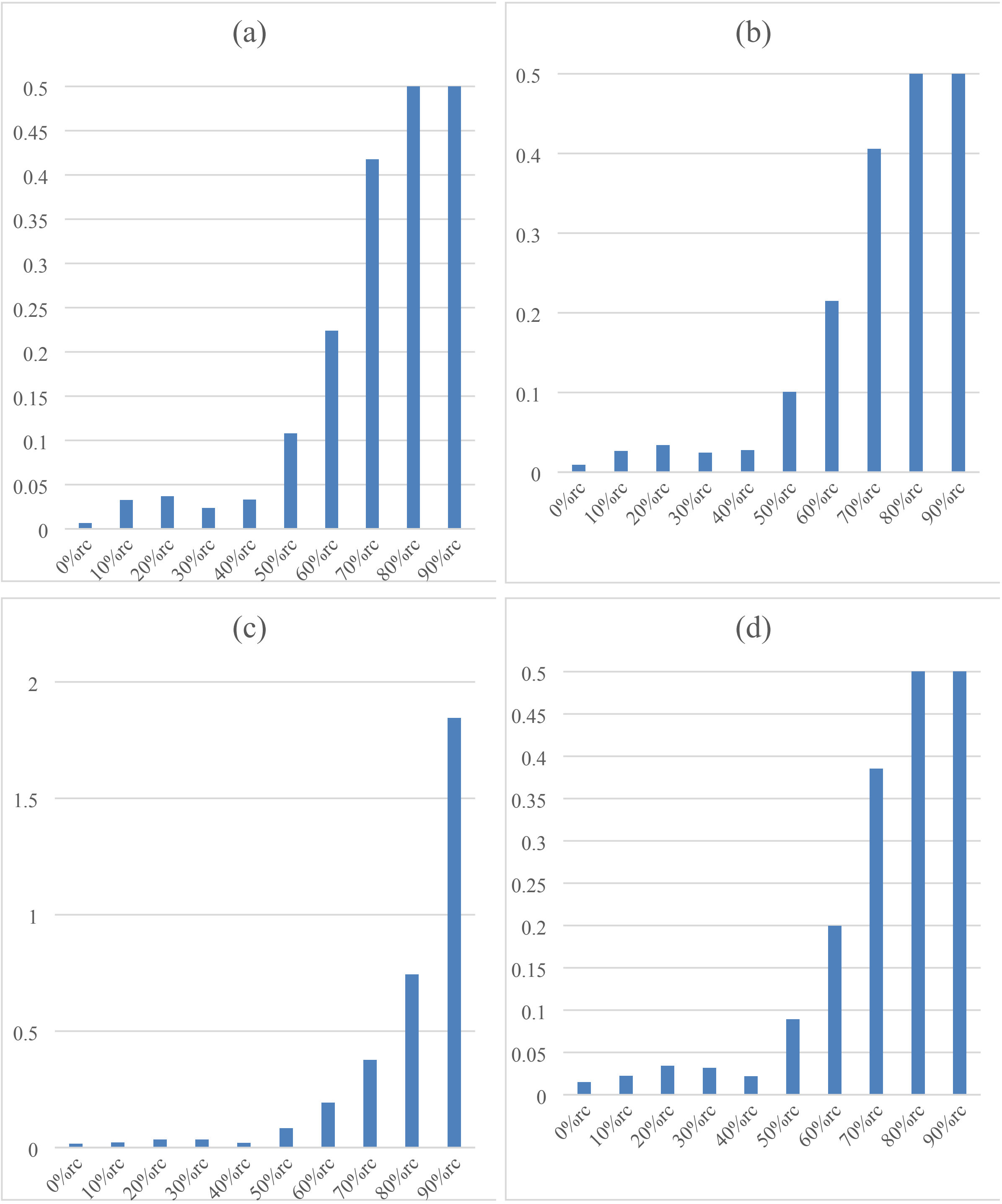

Please do not adjust margins

Please do not adjust margins

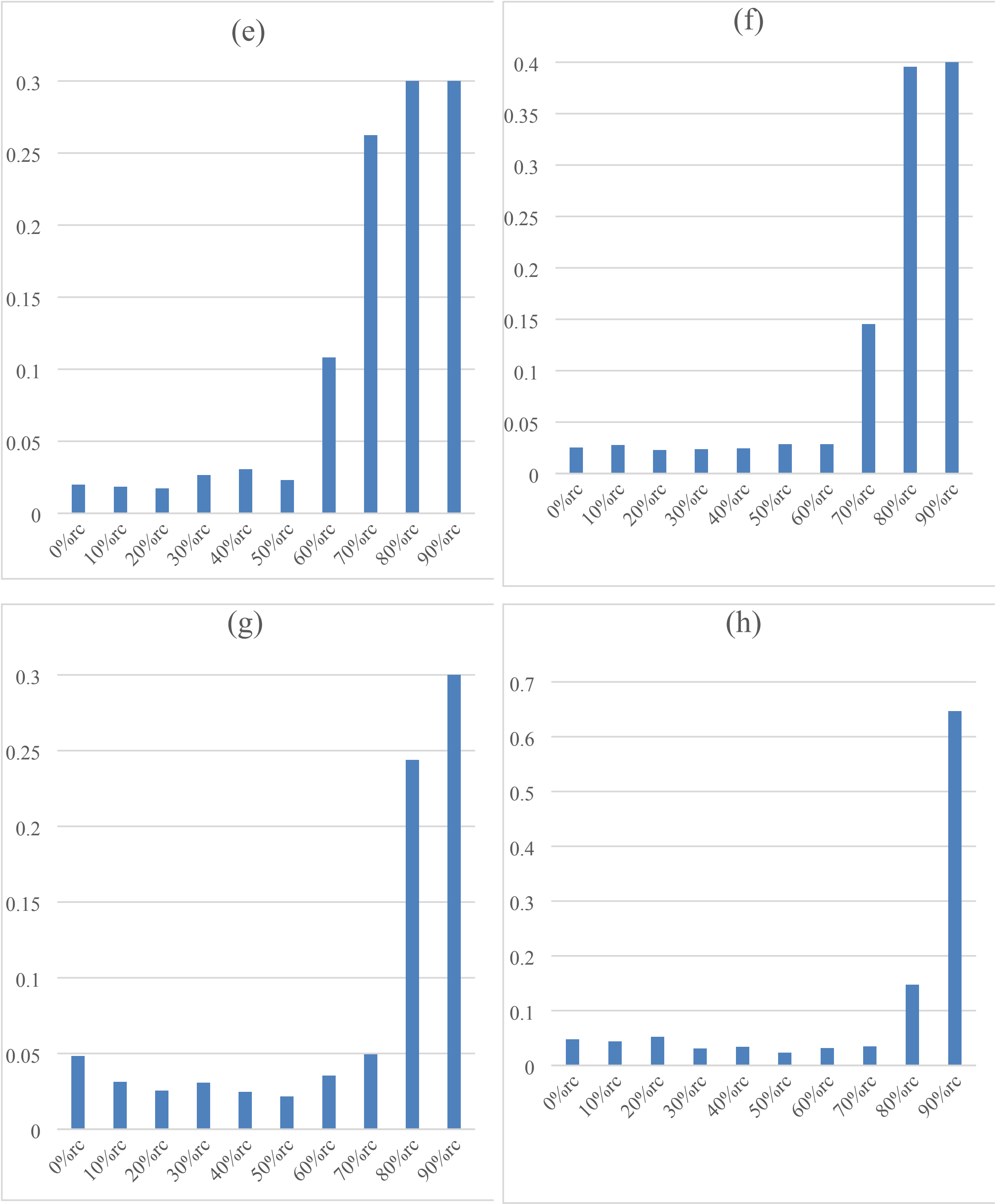

This journal is © The Royal Society of Chemistry 20xx

Please do not adjust margins

Please do not adjust margins

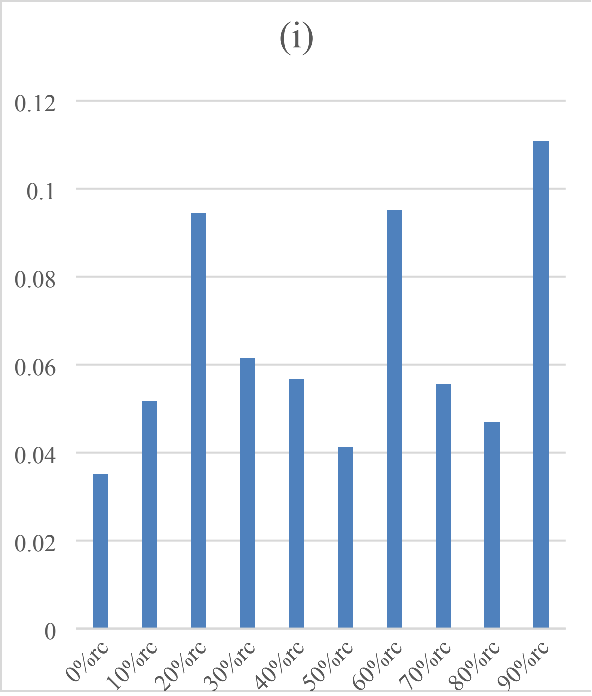

Figure S4. NRMSDs for the fitting of the BSA model spectrum to experimental data as a function of percentage RC removed from the original spectrum at (a) 20°C, (b) 30°C, (c) 40 °C, (d) 50°C,(e) 60°C, (f) 70°C, (g) 80°C, (h) 90°C, and (i) 100°C.

Please do not adjust margins

6. SOMSpec NRMSD output for lysozyme

Figures S5 shows the spectral NRMSDs of the best model spectrum from the derandomised experimental lysozyme spectra with varying percentage RC removed at each temperature.

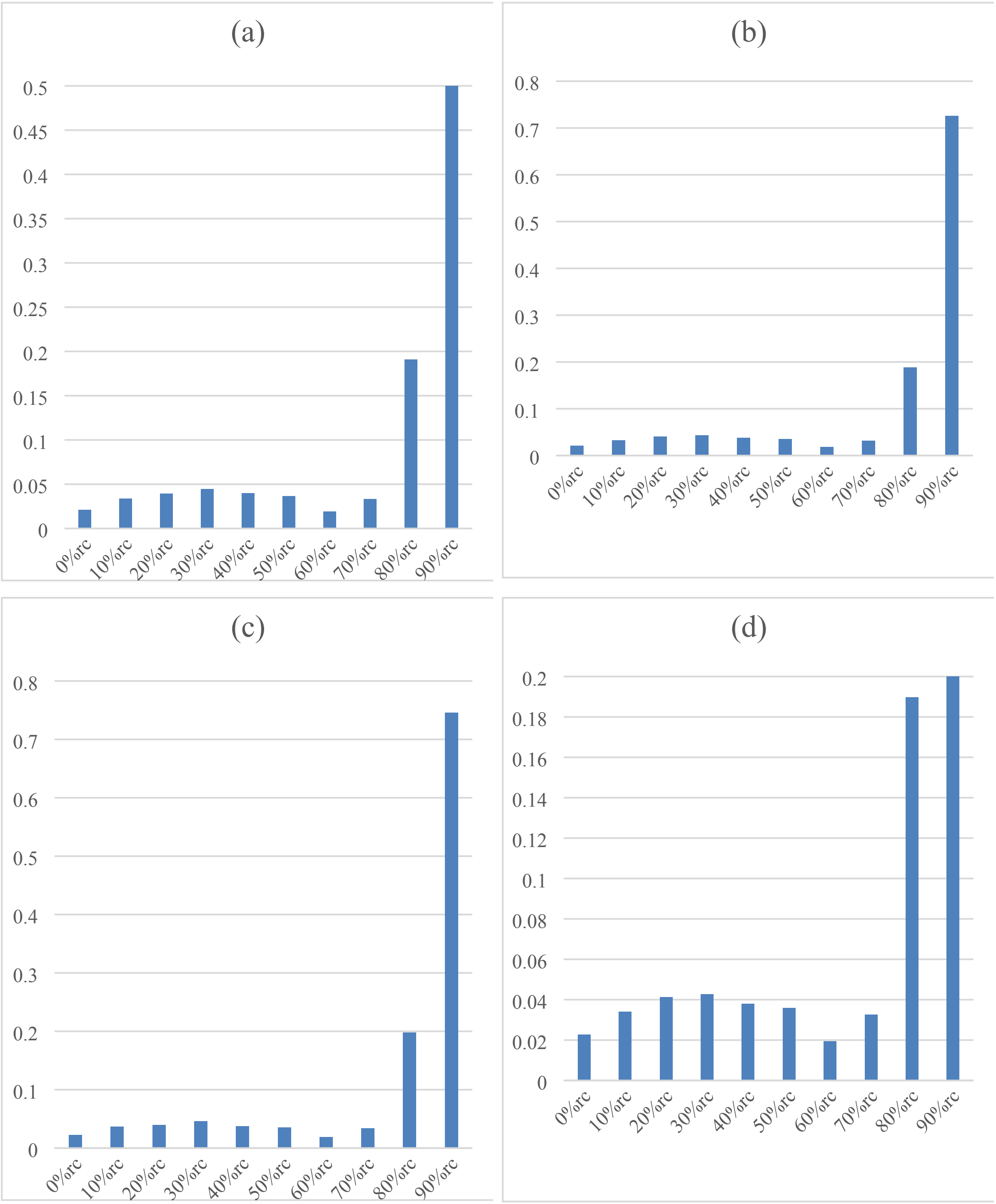

 This journal is © The Royal Society of Chemistry 20xx

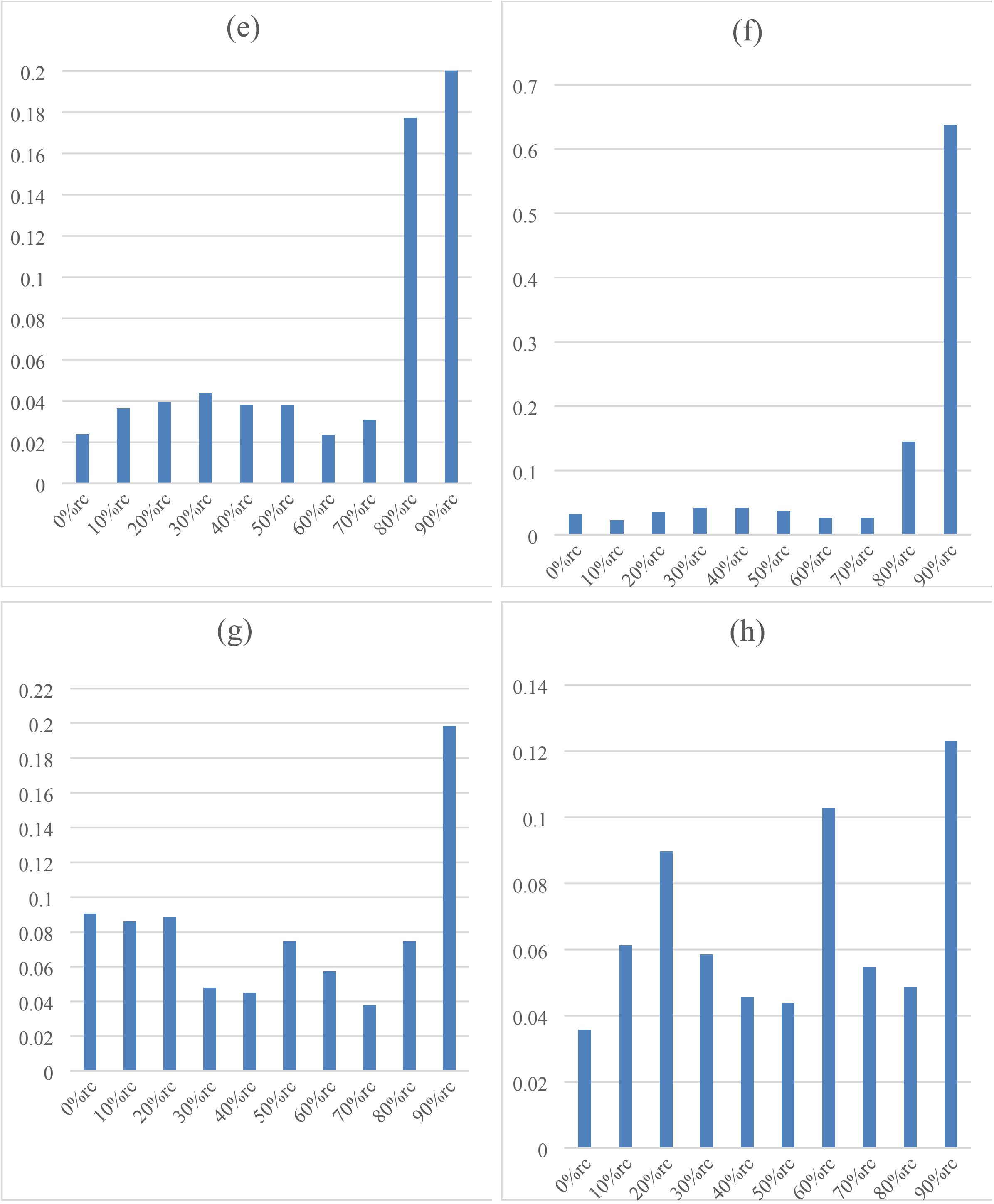
(e)
0.2
0.18
0.16
0.14
0.12
0.1
0.08
0.06
0.04
0.02
0
0%orc
10%orc
20%orc
30%orc
40%orc
50%orc
60%orc
70%orc
80%orc
90%orc
(f)
0.7
0.6
0.5
0.4
0.3
0.2
0.1
0
0%orc
10%orc
20%orc
30%orc
40%orc
50%orc
60%orc
70%orc
80%orc
90%orc
(g)
0.22
0.2
0.18
0.16
0.14
0.12
0.1
0.08
0.06
0.04
0.02
0
0%orc
10%orc
20%orc
30%orc
40%orc
50%orc
60%orc
70%orc
80%orc
90%orc
(h)
0.14
0.12
0.1
0.08
0.06
0.04
0.02
0
0%orc
10%orc
20%orc
30%orc
40%orc
50%orc
60%orc
70%orc
80%orc
90%orc

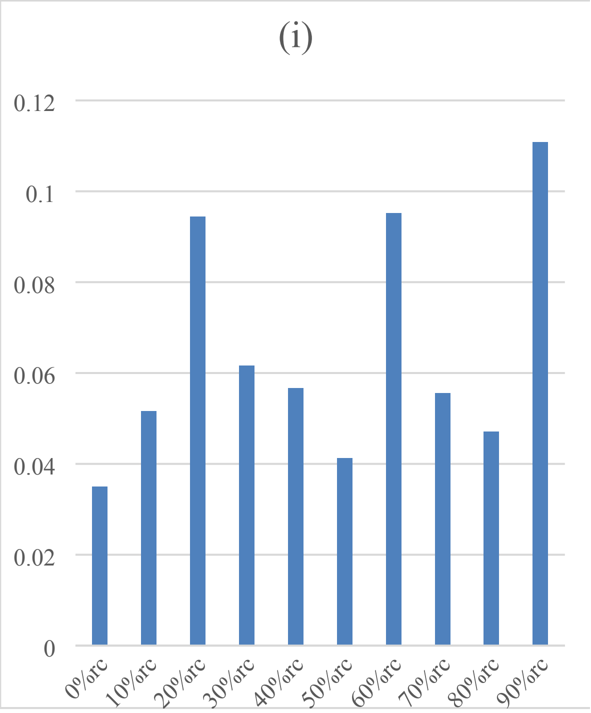

Figure S5. NRMSDs for the fitting of the lysozyme model spectrum to experimental data as a function of percentage RC removed from the original spectrum at (a) 20°C, (b) 30°C, (c) 40 °C, (d) 50°C,(e) 60°C, (f) 70°C, (g) 80°C, (h) 90°C, and (i) 100°C.

This journal is © The Royal Society of Chemistry 20xx

Please do not adjust margins

7. SOMSpec NRMSD output for insulin

Figures S6 shows the spectral NRMSDs of the best model spectrum from the derandomised experimental insulin spectra with varying percentage RC removed at each temperature.

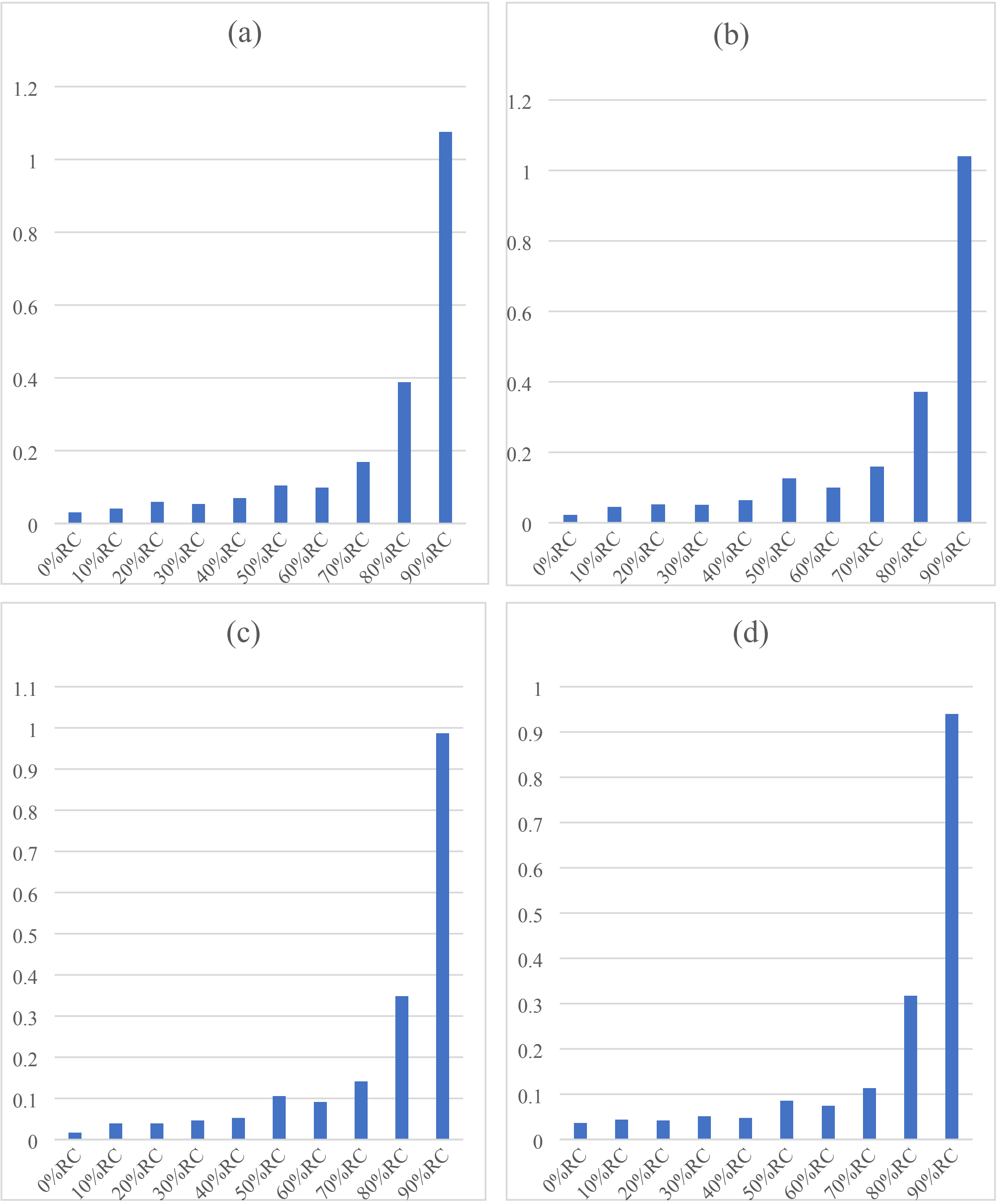

This journal is © The Royal Society of Chemistry 20xx

Please do not adjust margins

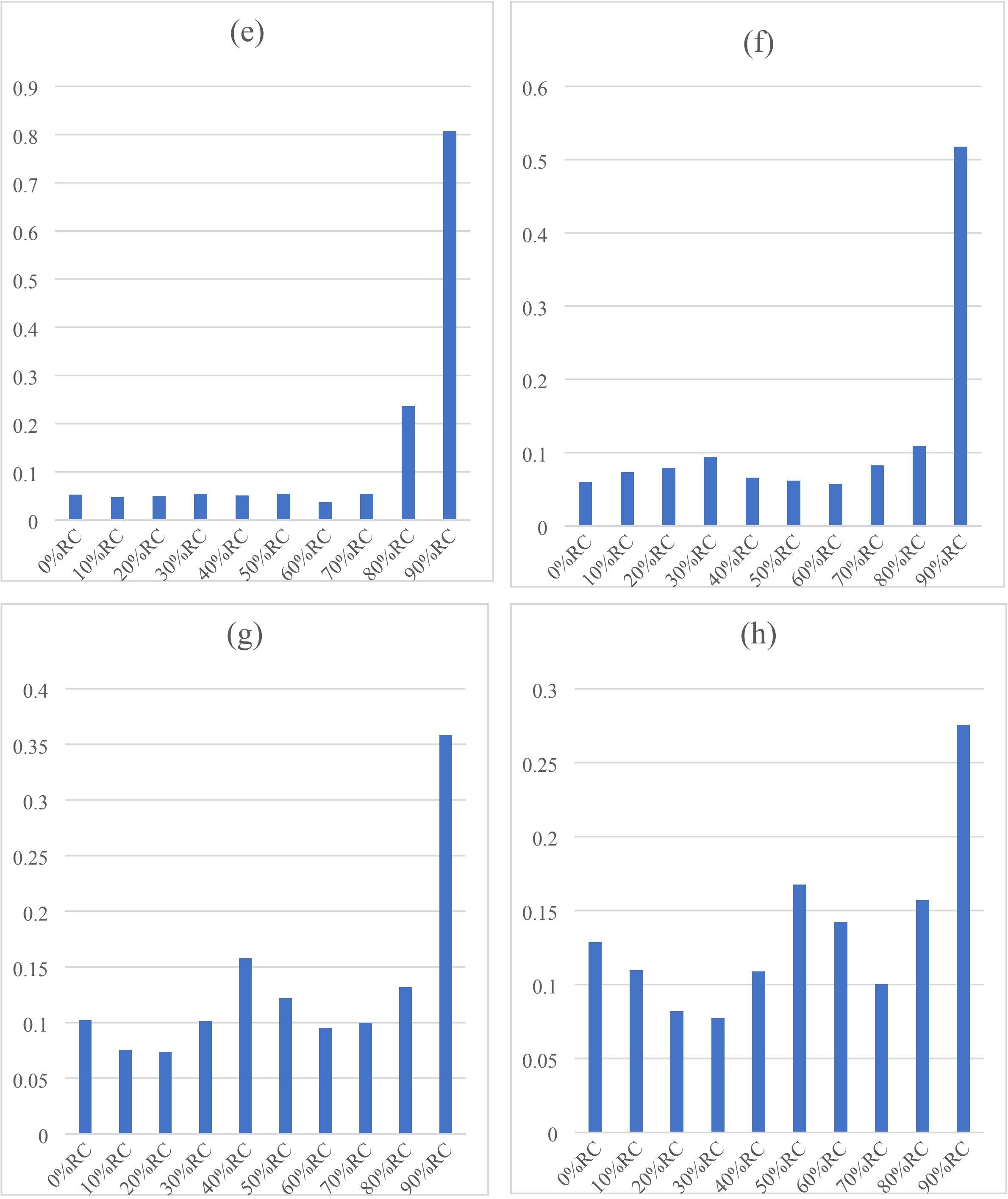

This journal is © The Royal Society of Chemistry 20xx

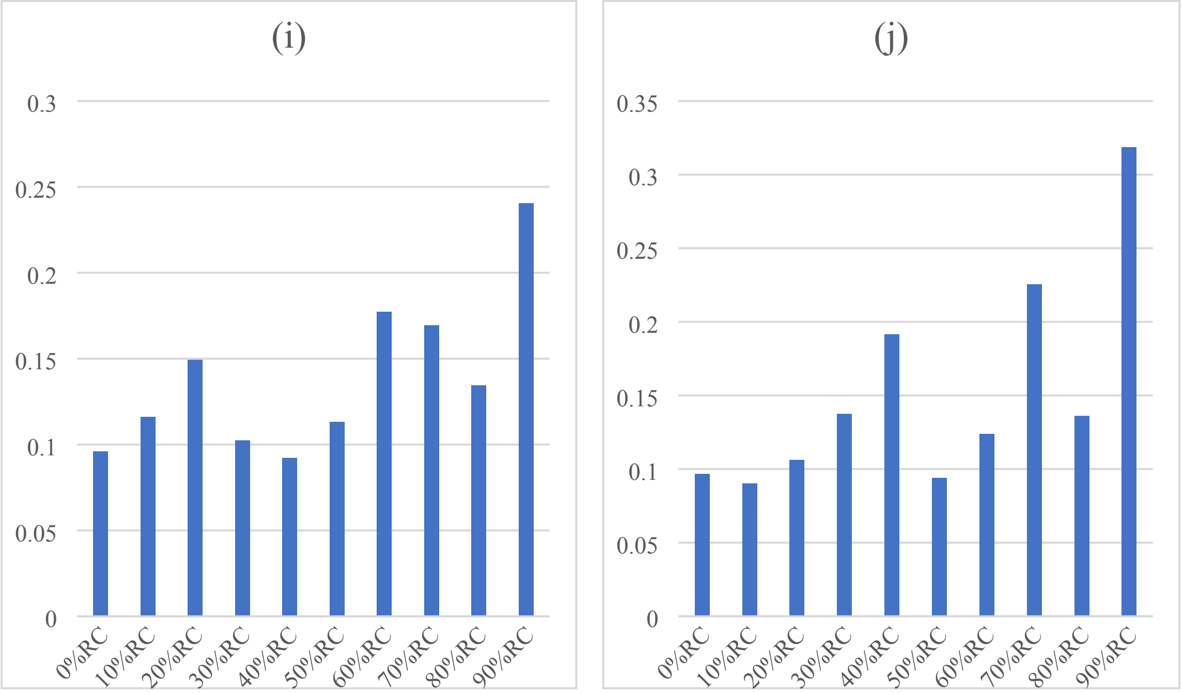

Figure S6. NRMSDs for the fitting of the insulin model spectrum to experimental data as a function of percentage RC removed from the original spectrum at (a) 20°C, (b) 30°C, (c) 40 °C, (d) 50°C,(e) 60°C, (f) 70°C, (g) 80°C, (h) 90°C, (i) 100°C, and(i)110°C.

8. SOMSpec model and experimental spectra for BSA

Figure S7 shows the overlay of the model specta and experimental spectra for BSA for each fraction of RC removed over the temperature range of 20 °C to 100°C in 10°C steps.

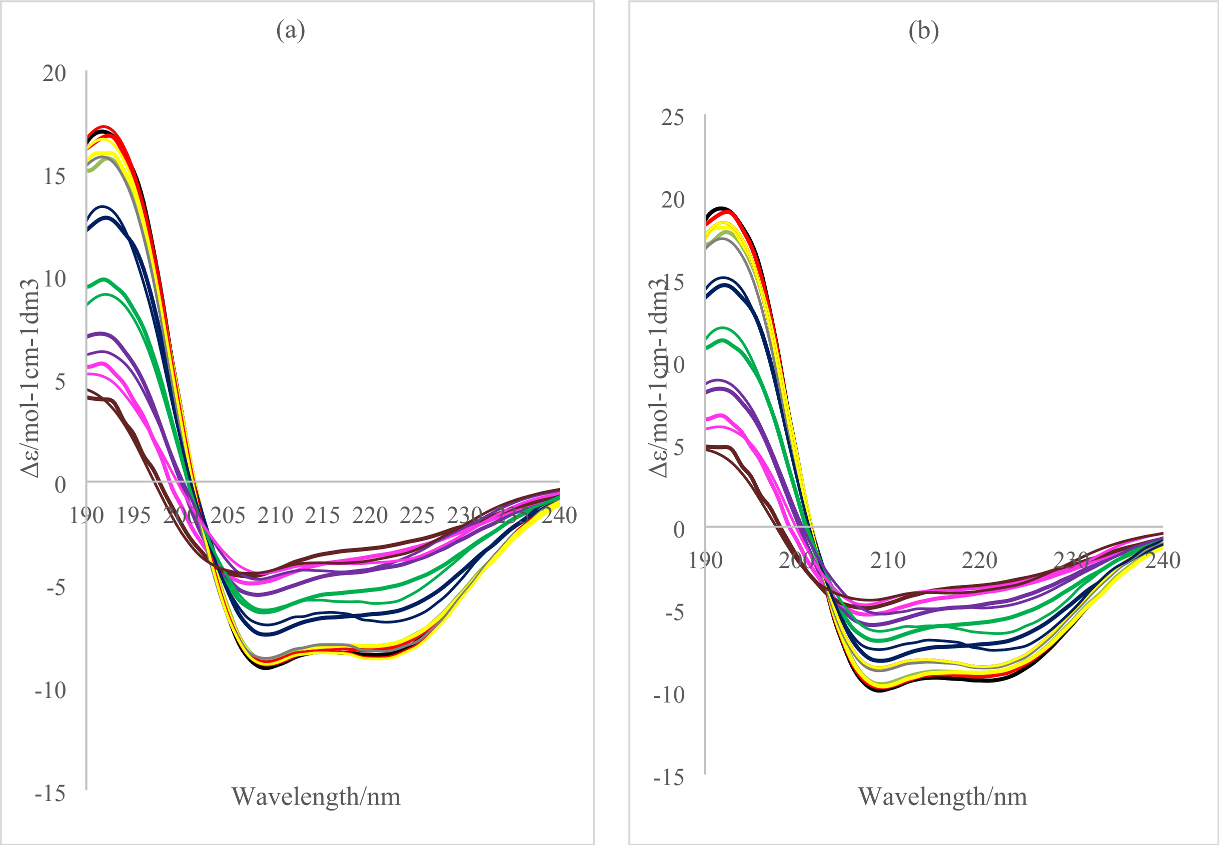

 This journal is © The Royal Society of Chemistry 20xx

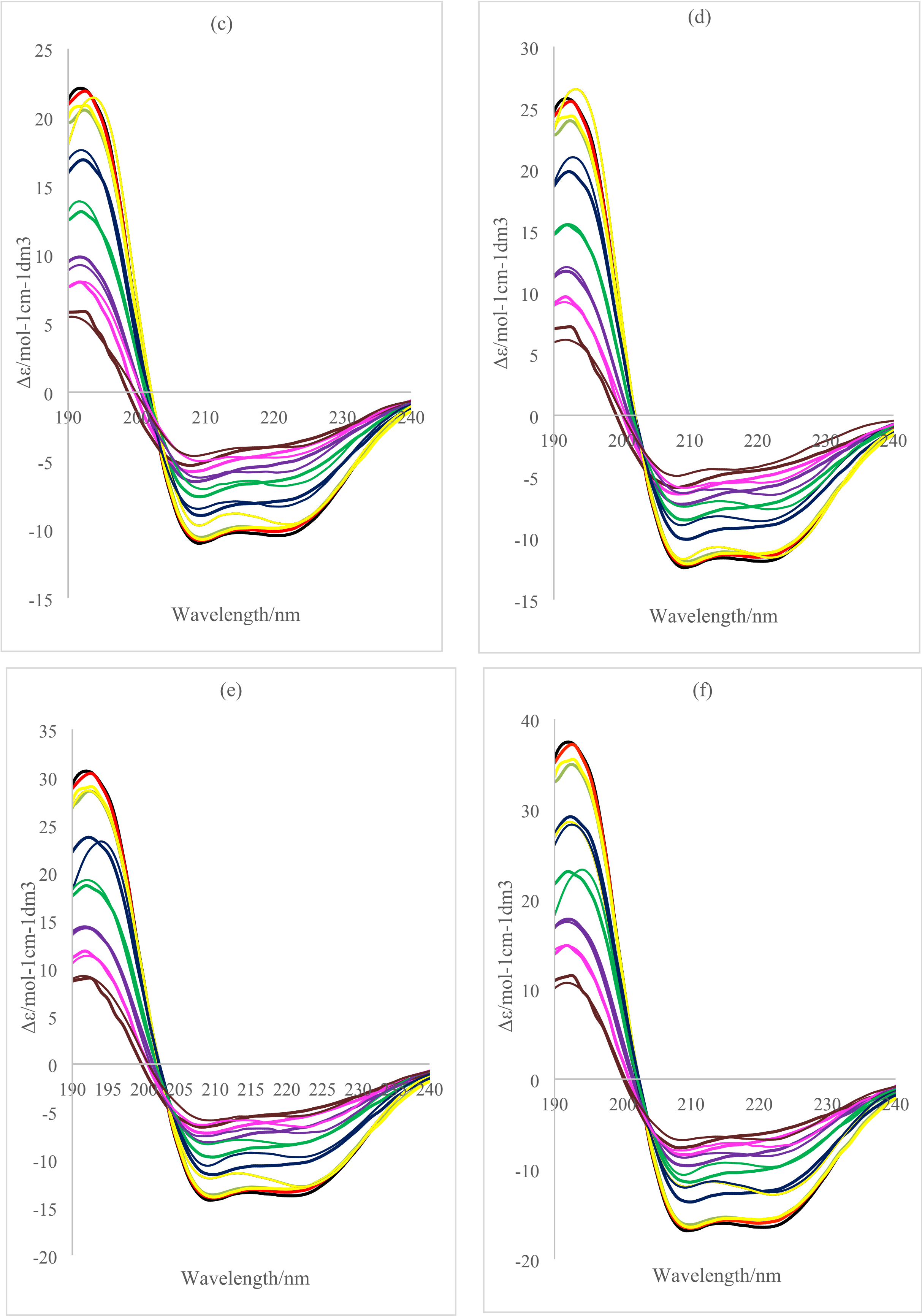

This journal is © The Royal Society of Chemistry 20xx

Please do not adjust margins

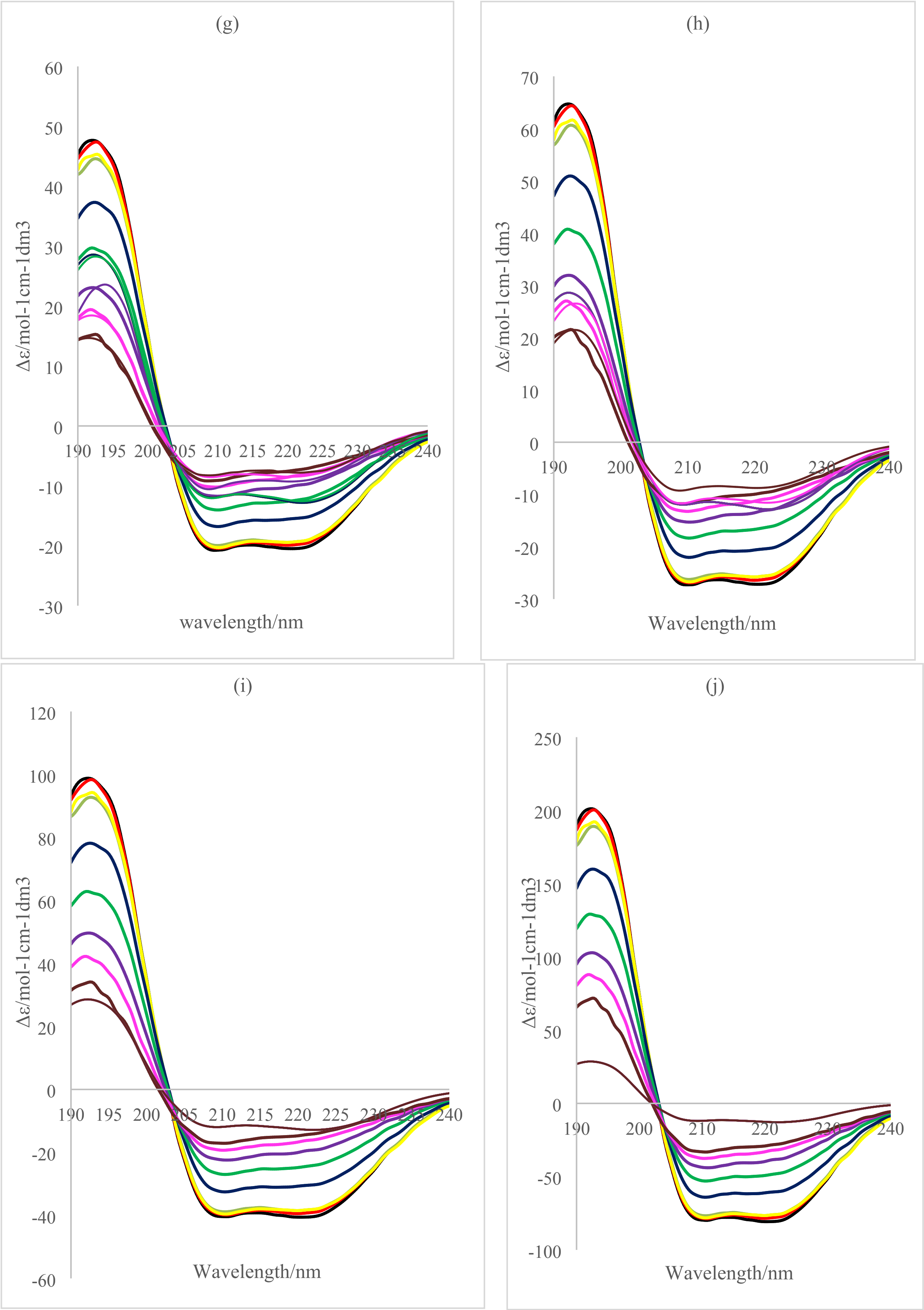
(g)
Δε/mol-1cm-1dm3
60
50
40
30
20
10
0
-10
-20
-30
190 195 200 205 210 215 220 225 230 235 240
wavelength/nm
(h)
Δε/mol-1cm-1dm3
70
60
50
40
30
20
10
0
-10
-20
-30
190 200 210 220 230 240
Wavelength/nm
(i)
Δε/mol-1cm-1dm3
120
100
80
60
40
20
0
-20
-40
-60
190 195 200 205 210 215 220 225 230 235 240
Wavelength/nm
(j)
Δε/mol-1cm-1dm3
250
200
150
100
50
0
-50
-100
190 200 210 220 230 240
Wavelength/nm

Please do not adjust margins

Figure S7. Overlay of BSA model and derandomised experimental spectra for (a) 0% RC, (b) 10% RC, (c) 20% RC, (d) 30% RC, (e) 40% rc, (f) 50% RC, (g) 60% RC, (h)70% RC, (i) 80% RC, (j) 90% RC. 20°C (black), 30°C (red), 40°C (grey), 50°C (yellow), 60°C (dark blue), 70°C (green), 80°C (purple), 90°C (pink), 100°C (dark orange). Unbroken and broken lines represent derandomised experimental and predicted spectra respectively.

This journal is © The Royal Society of Chemistry 20xx

Please do not adjust margins

9. SOMSpec model and experimental spectra for lysozyme

Figure S8 shows the overlay of the model specta and experimental spectra for lysozyme for each fraction of RC removed over the temperature range of 20 °C to 100°C in 10°C steps.

.

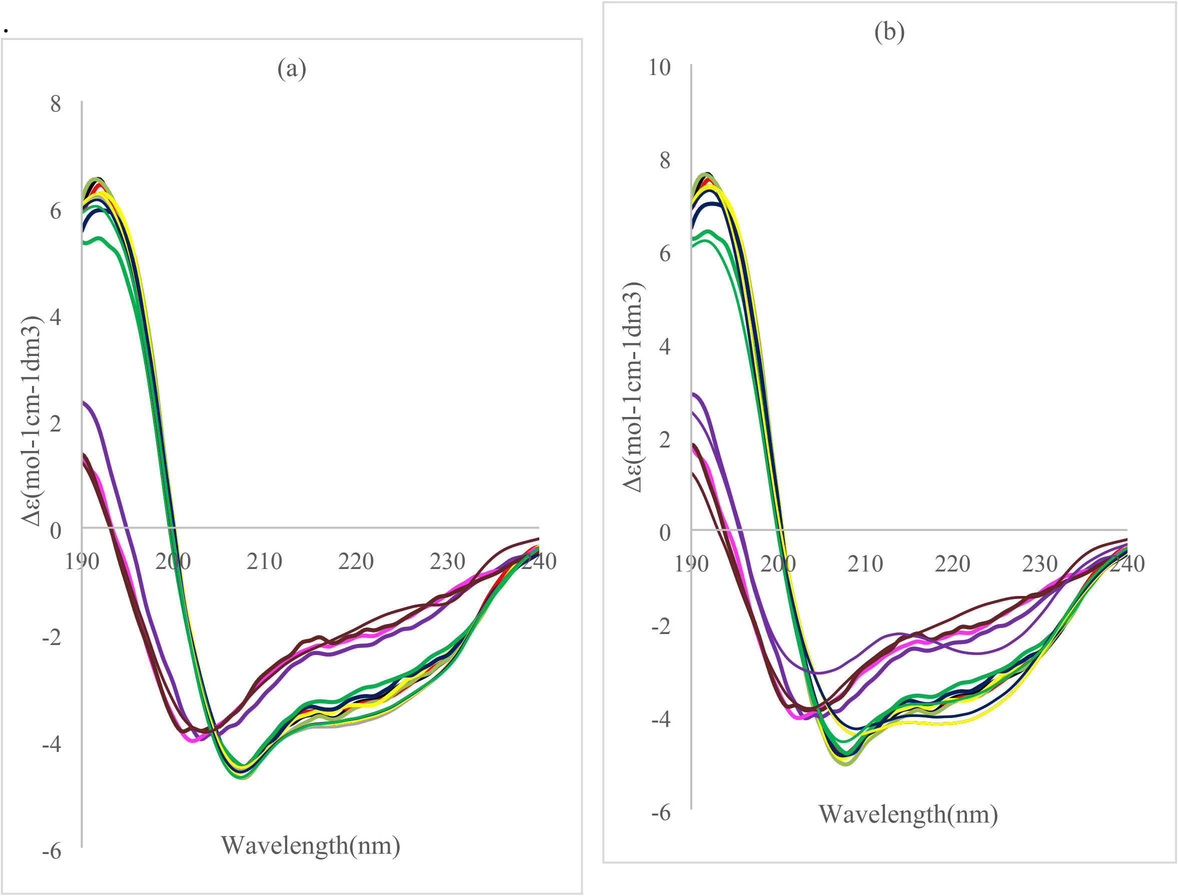

This journal is © The Royal Society of Chemistry 20xx

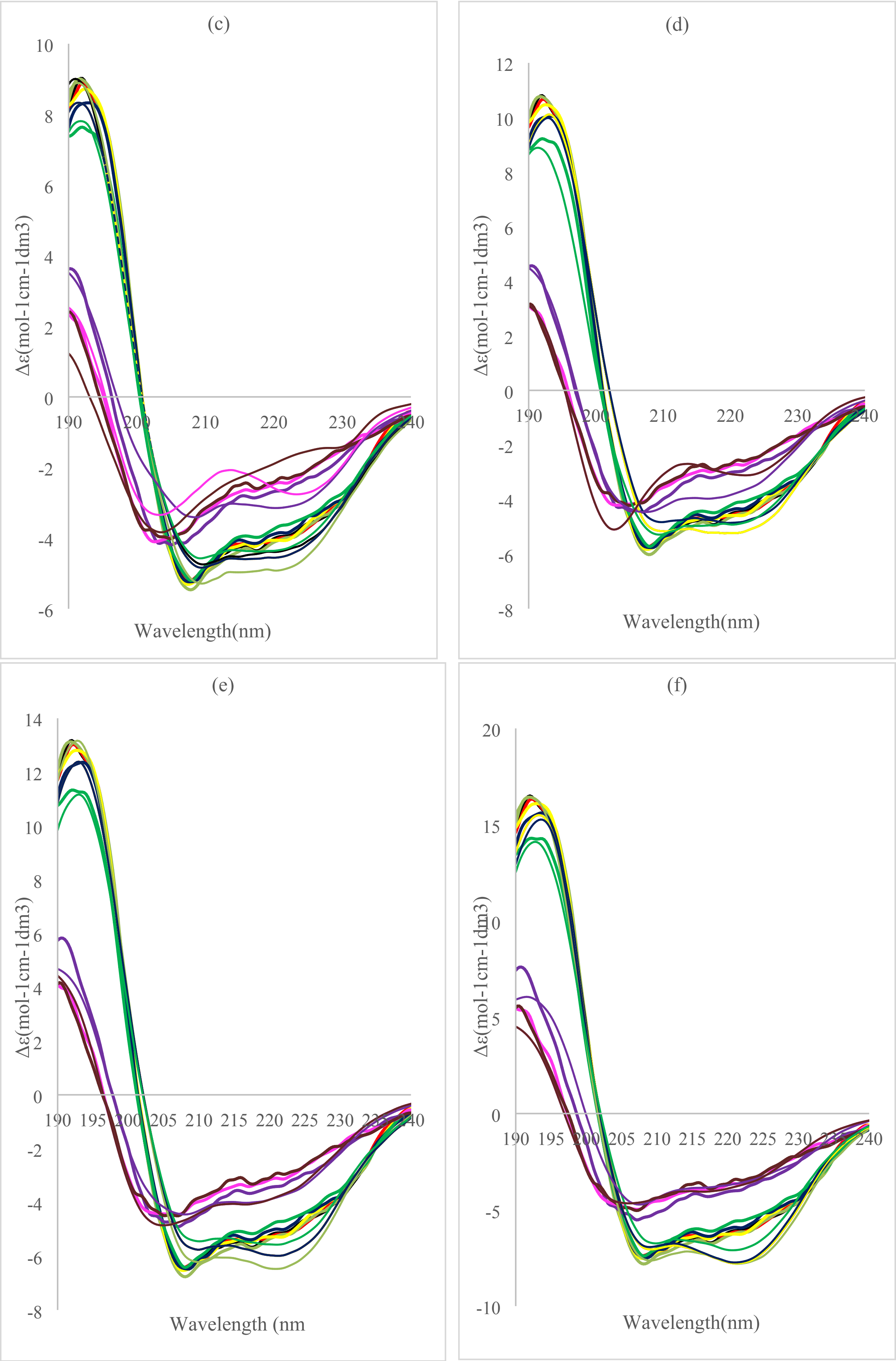
(c)
Δε(mol-1cm-1dm3)
Wavelength(nm)
(d)
Δε(mol-1cm-1dm3)
Wavelength(nm)
(e)
Δε(mol-1cm-1dm3)
Wavelength (nm
(f)
Δε(mol-1cm-1dm3)
Wavelength(nm)

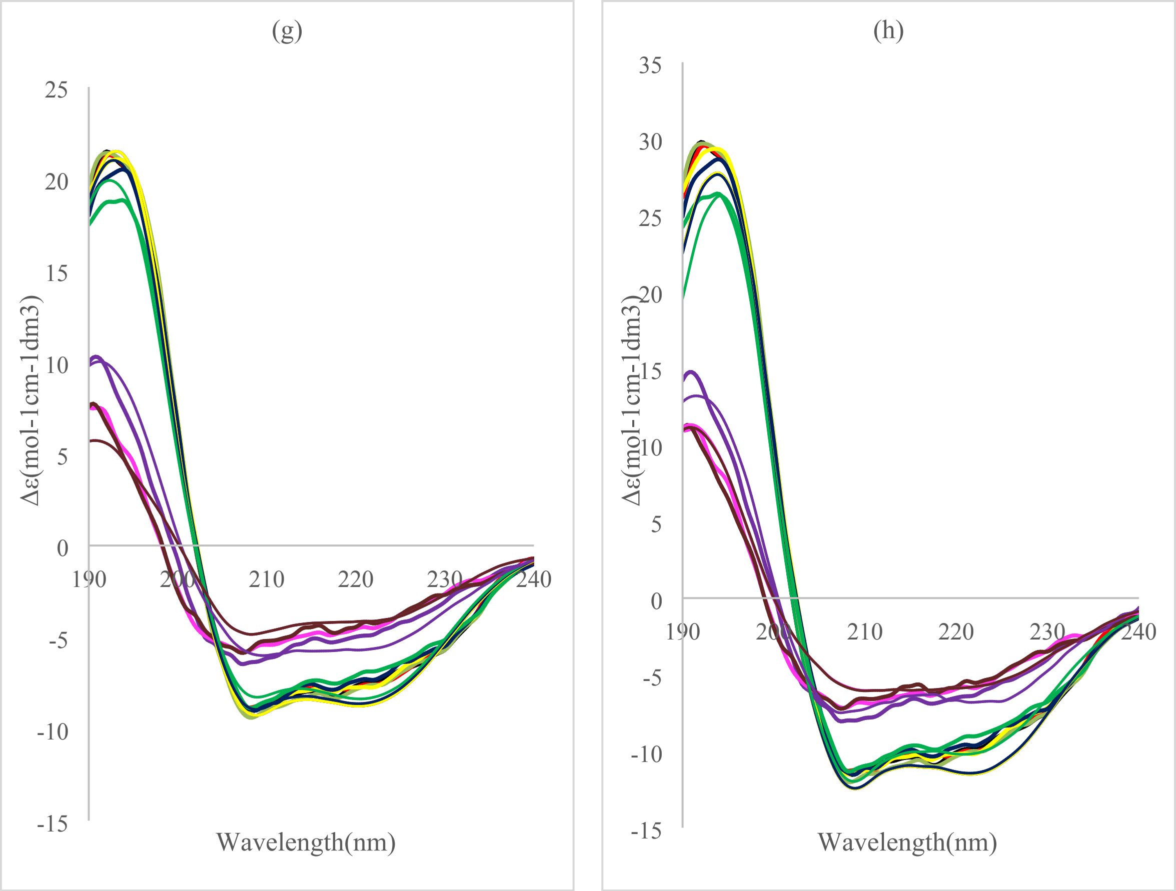

This journal is © The Royal Society of Chemistry 20xx

Please do not adjust margins

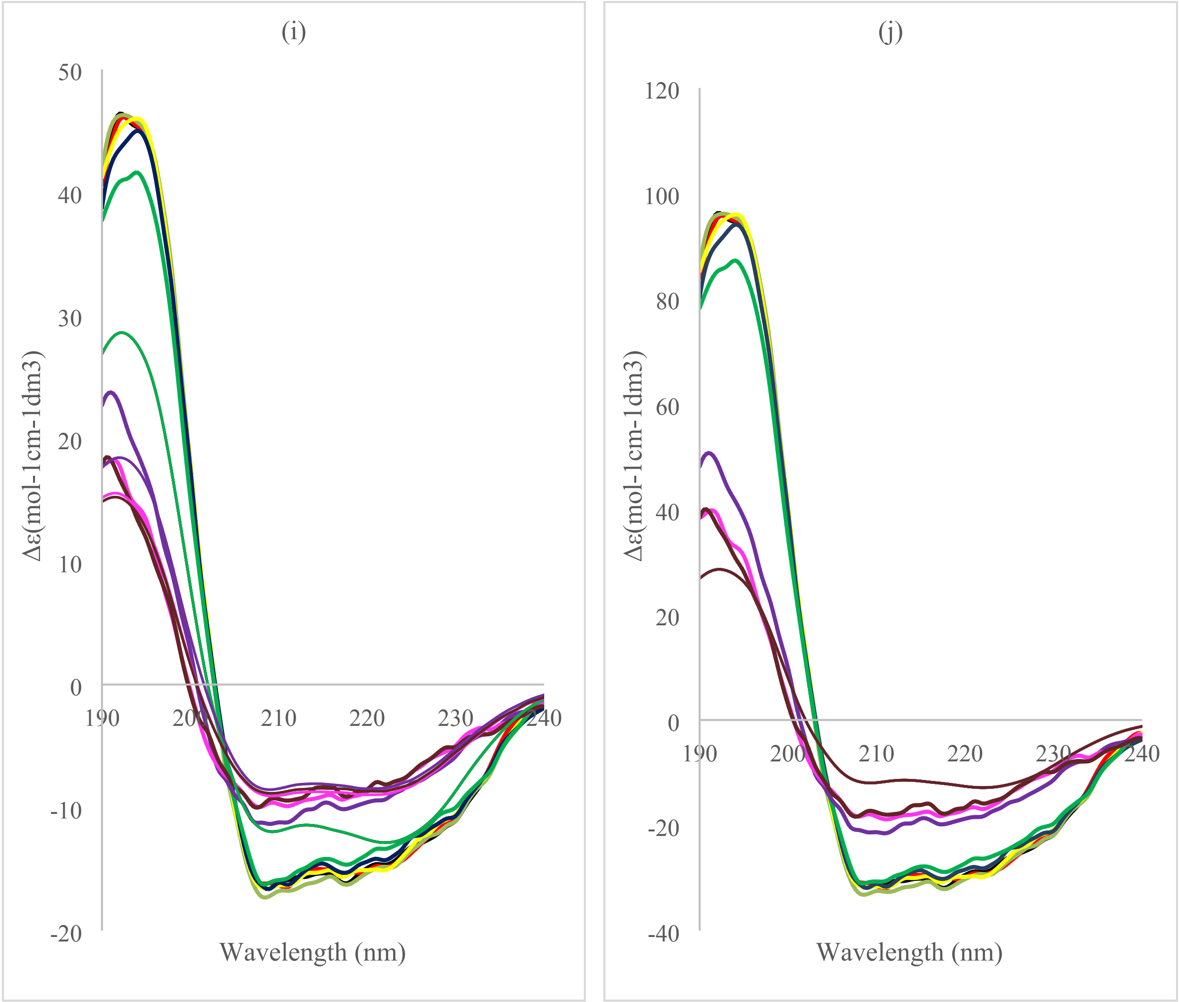

Figure S8. Overlay of lysozyme model and derandomised experimental spectra for (a) 0% RC, (b) 10% RC, (c) 20% RC, (d) 30% RC, (e) 40% rc, (f) 50% RC, (g) 60% RC, (h)70% RC, (i) 80% RC, (j) 90% RC. 20°C (black), 30°C (red), 40°C (grey), 50°C (yellow), 60°C (dark blue), 70°C (green), 80°C (purple), 90°C (pink), 100°C (dark orange). Unbroken and broken lines represent derandomised experimental and predicted spectra respectively.

This journal is © The Royal Society of Chemistry 20xx

Please do not adjust margins

Please do not adjust margins

10. SOMSpec model and experimental spectra for insulin

Figure S9 shows the overlay of the model spectra and experimental spectra for insulin for each fraction of RC removed over the temperature range of 20 °C to 110°C in 10°C steps.

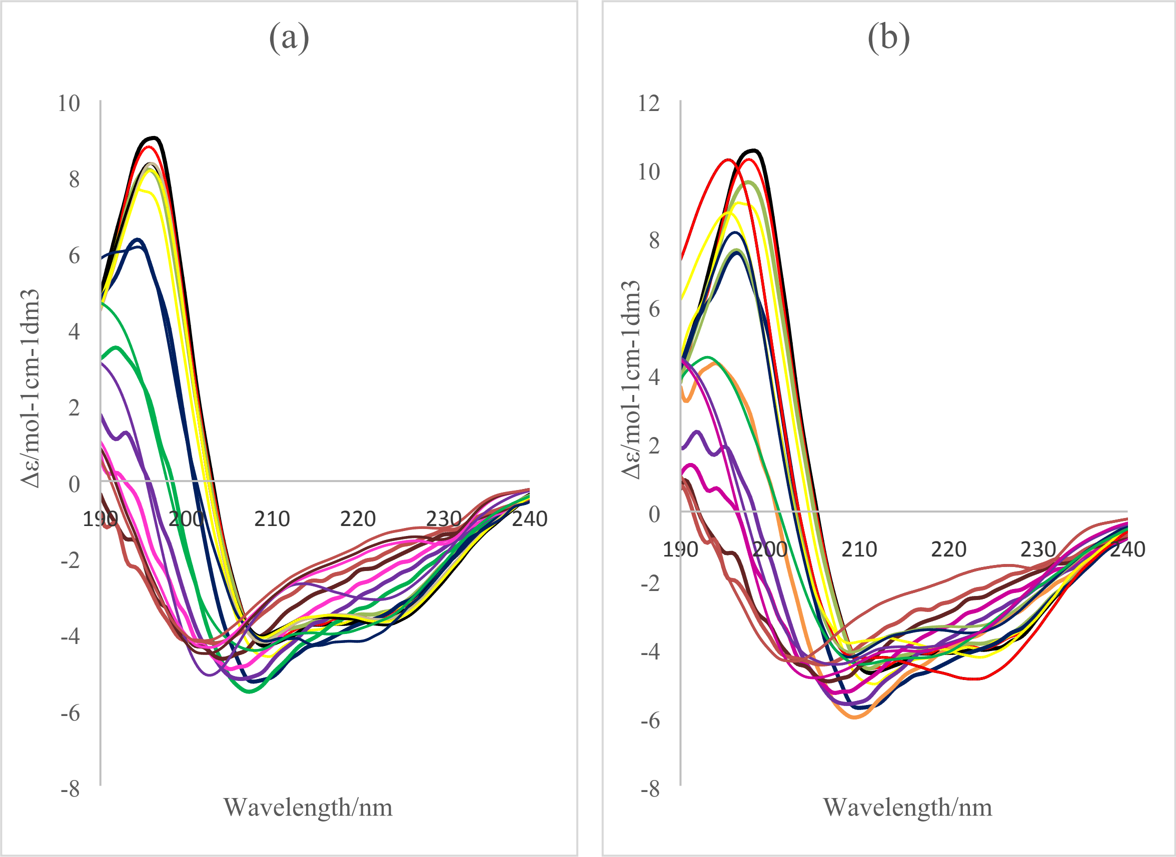

This journal is © The Royal Society of Chemistry 20xx

Please do not adjust margins

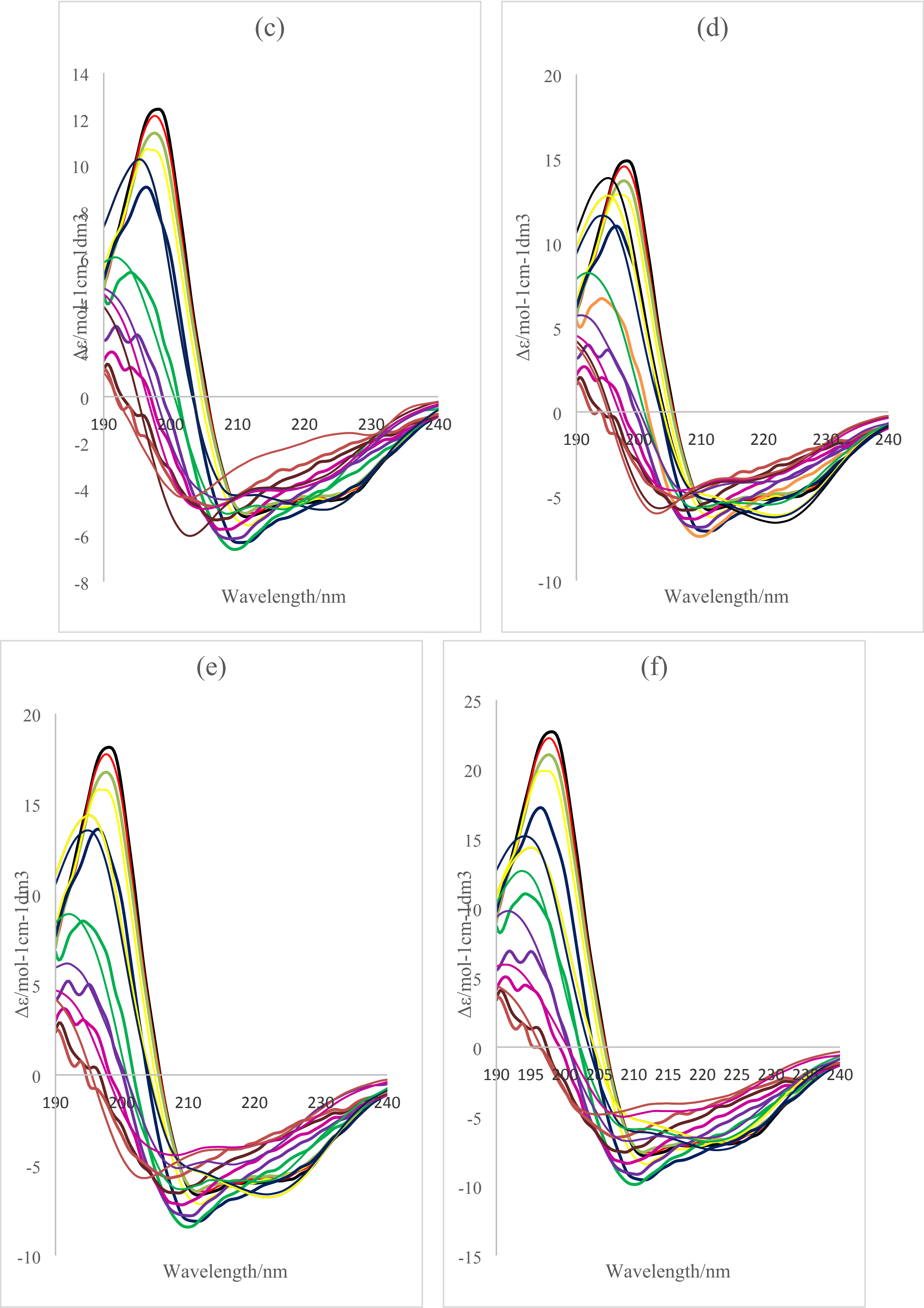
(c)
14
12
10
8
6
4
2
0
-2
-4
-6
-8
190
200
210
220
230
240
Δε/mol-1cm-1dm3
Wavelength/nm
(d)
20
15
10
5
0
-5
-10
190
200
210
220
230
240
Δε/mol-1cm-1dm3
Wavelength/nm
(e)
20
15
10
5
0
-5
-10
190
200
210
220
230
240
Δε/mol-1cm-1dm3
Wavelength/nm
(f)
25
20
15
10
5
0
-5
-10
-15
190 195 200 205 210 215 220 225 230 235 240
Δε/mol-1cm-1dm3
Wavelength/nm

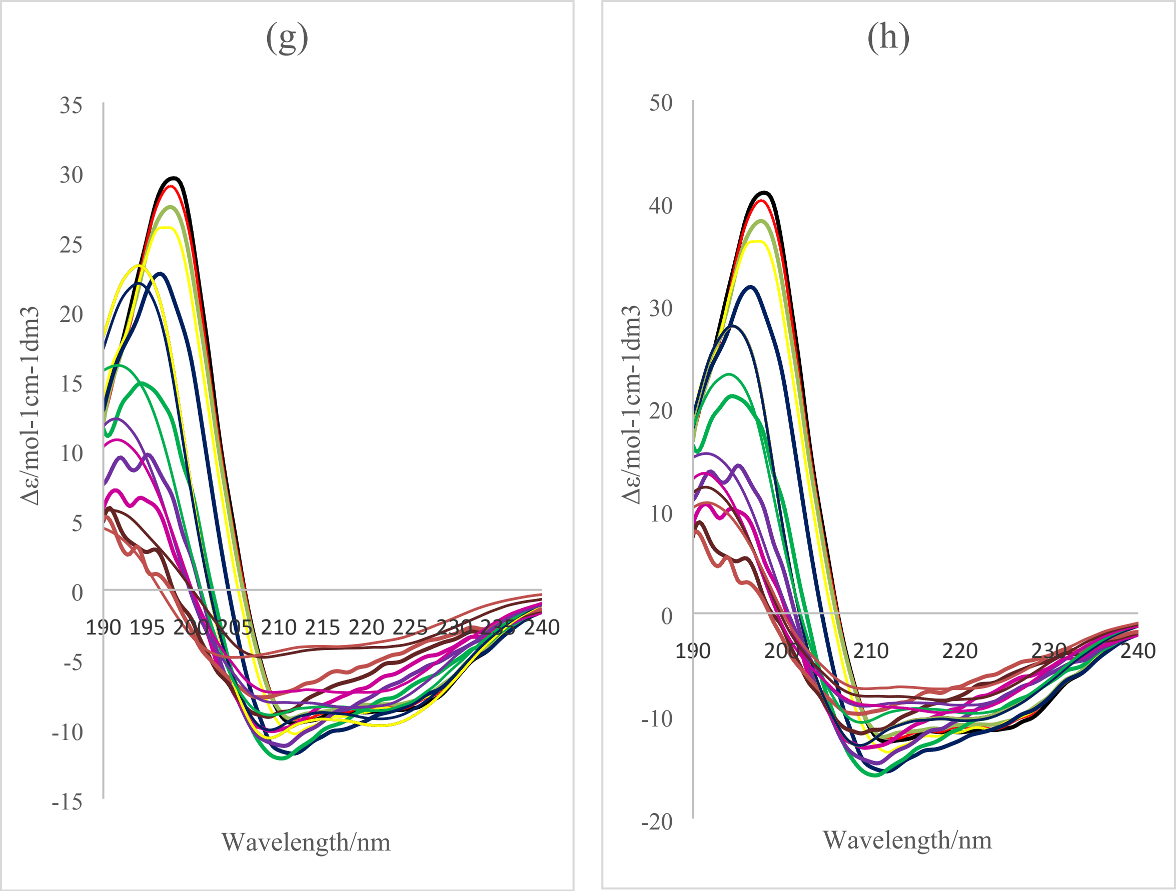

 This journal is © The Royal Society of Chemistry 20xx

Please do not adjust margins

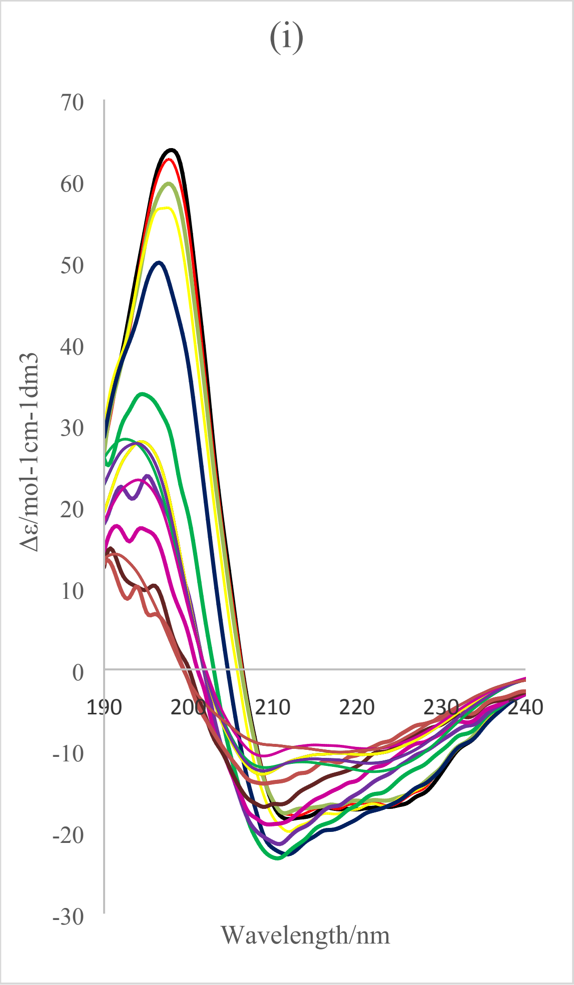

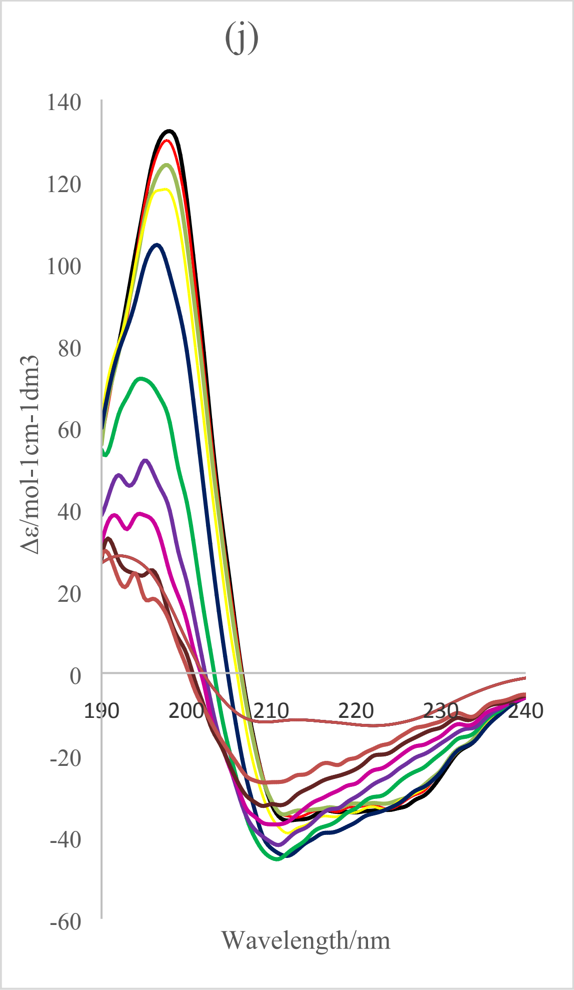

Figure S9. Overlay of insulin model and derandomised experimental spectra for (a) 0% RC, (b) 10% RC, (c) 20% RC, (d) 30% RC, (e) 40% rc, (f) 50% RC, (g) 60% RC, (h)70% RC, (i) 80% RC, (j) 90% RC. 20°C (black), 30°C (red), 40°C (grey), 50°C (yellow), 60°C (dark blue), 70°C (green), 80°C (purple), 90°C (pink), 100°C (dark orange), 110 ^{o}C (orange). Unbroken and broken lines represent derandomised experimental and predicted spectra respectively.

Please do not adjust margins

Please do not adjust margins

11. Procedure for preparing input and summarising output for SOMSpec

This standard operating procedure is designed to guide the user through a series of MATLAB units to prepare input data for SOMSpec, to use SOMSpec and to extract the SOMSpec output into a user-friendly format. This set of instructions is written in terms of a reference set of spectra data from 240–190 nm in 1 nm increments, a random coil spectrum from 240–190 nm in 1 nm increments, and an xls file with spectral data from 190–260 nm in 0.2 nm increments. YOU COULD PUT ASPECTS OF YOUR PARAGRAPH HERE AS A SUMMARY (but see below first).

Procedure

1) Make sure the step size of the data is 1 nm, and its wavelength range is between 240–190 nm. If it is not, for example, if the spectra run from 190–260 nm in 0.2 nm steps:
    a. Invert the data by sorting it by wavelength from highest to lowest in Excel, using the sort and filter group in the data tab.
    b. Cut the first 20 nm to give a range starting from 240 nm.
    c. Assuming the wavelength data step size is 0.2 nm, delete four data points in between the whole number wavelengths such as 240 and 239, 239 and 238,…, 191 and 190 nm to give a 51 element spectrum. This can be done in various ways. If column A has the wavelengths and 240 nm starts at position A2, then one method is to create a column based on if(int(A#)=A#, A#,0). This will then generate a column with integers at each whole wavelength and a 0 for all the others. Now sort highest to lowest by that column and only retain the top 52 (including column label) rows.

2) Save the input files as xls spreadsheet (Excel 97-2003 Workbook) in a folder named "New folder" for the CD/UV/IR and delta epsilon modules of the **CDspectra** app ~~and as a formatted txt for the SOMSPEC app.~~ Ensure these xls files have column labels in the first row for each spectrum, wavelengths in the first column, a baseline spectrum (or zeros) in column 2, and spectra in subsequent columns. The raw CD spectra for the example provided for BSA are with column labels ending with a number from (1 to 9) indicating 9 spectra collected as a function of temperature from (20 ^{o}C to 100°C in 10 ^{o}C increments).

3) The disordered module xls input files must also be with column labels; its first, second, subsequent columns correspond to wavelengths, standard random spectrum, and spectra, respectively.

4) Double-click on the app installer (**CDspectra**) to start Matlab R2019b, and then click install.

 This journal is © The Royal Society of Chemistry 20xx

Please do not adjust margins

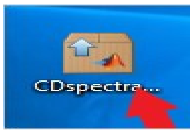

5) Click on the APPS tab on the top right corner of Matlab R2019, and click CDspectra to launch the app.

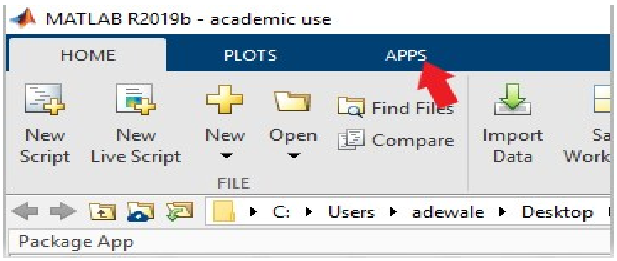

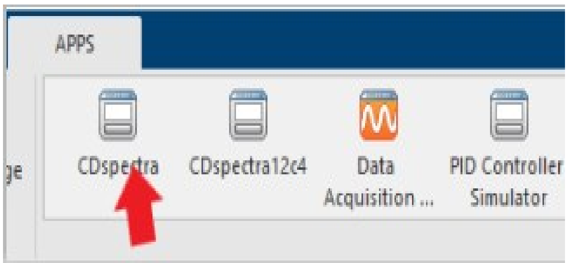

6) Change the working directory to the folder where the input files are in. *E.g.,* if they are in New folder, click on the browse for folder icon, then **New folder** and follow by **Select Folder** to insert the specified working directory.

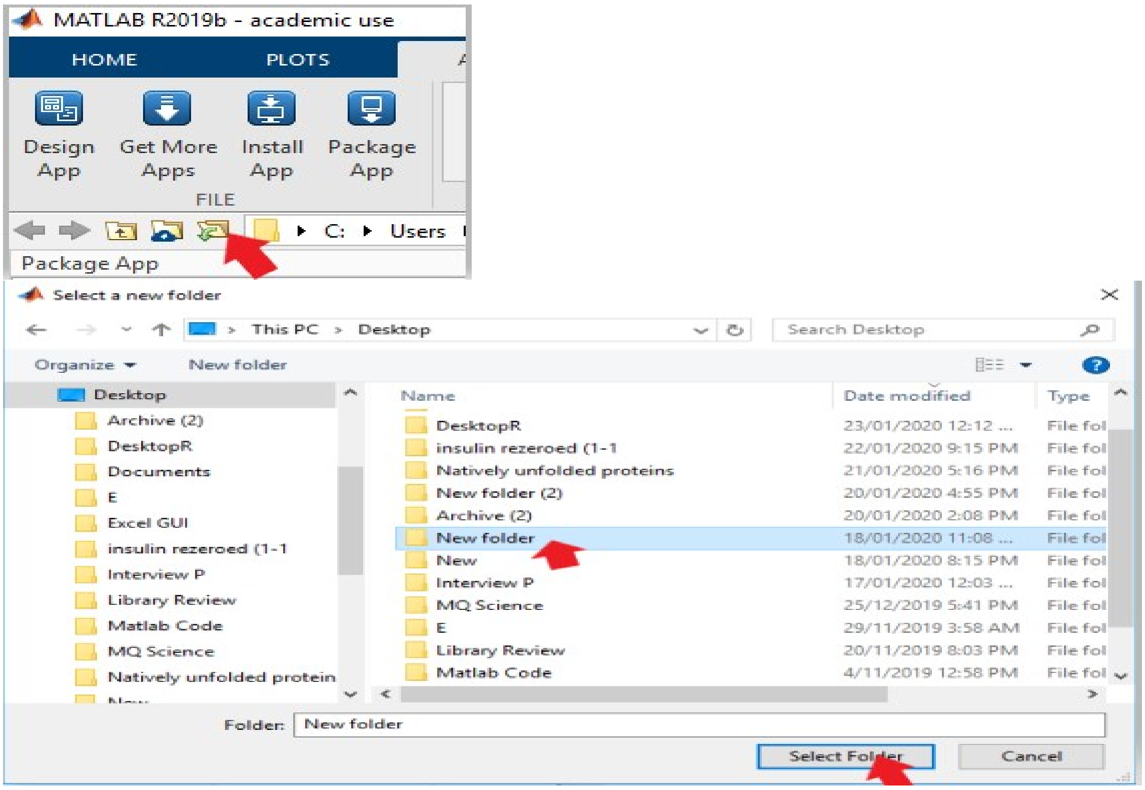

7) Click on the **CD/UV/IR** module of the app to baseline correct and plot the raw CD spectra of BSA. Then, hit the "**select**" push button to select an appropriate excel file (*e.g.,* the BSA file **BSA Tm wavelength_ch1_CD(mdeg)**). Follow by clicking on the "Baseline corrected" button to generate the baseline corrected spectra (determined by subtracting column 2 from every subsequent spectrum), and specify spectroscopy type as a CD. The output xls file is called "**BCMDBSA Tm**

Please do not adjust margins

wavelength_ch1_CD(mdeg),” where BCMD is an acronym for baseline corrected millidegrees. You may need to click on the operating window or the file list windows to make them on top.

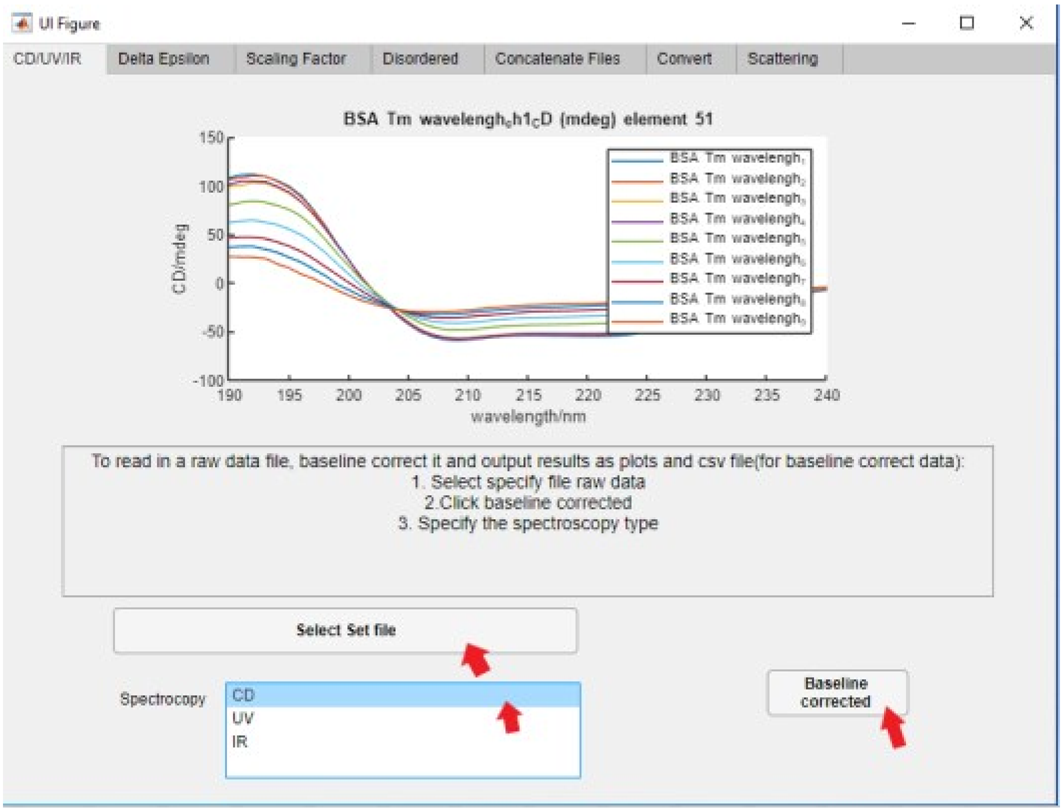

8) Switch to the **Delta Epsilon** module of the app to convert spectra from millidegrees to delta epsilon. Specify the values of the input parameters by typing the things I have typed on the screenshot below, then click on the “baseline corrected” push button to generate its corresponding plot and xls file. The output xls file is called “**BCDEBSA Tm wavelength_ch1_CD(mdeg)**,” where BCDE is an acronym for baseline corrected delta epsilon.

This journal is © The Royal Society of Chemistry 20xx
Please do not adjust margins

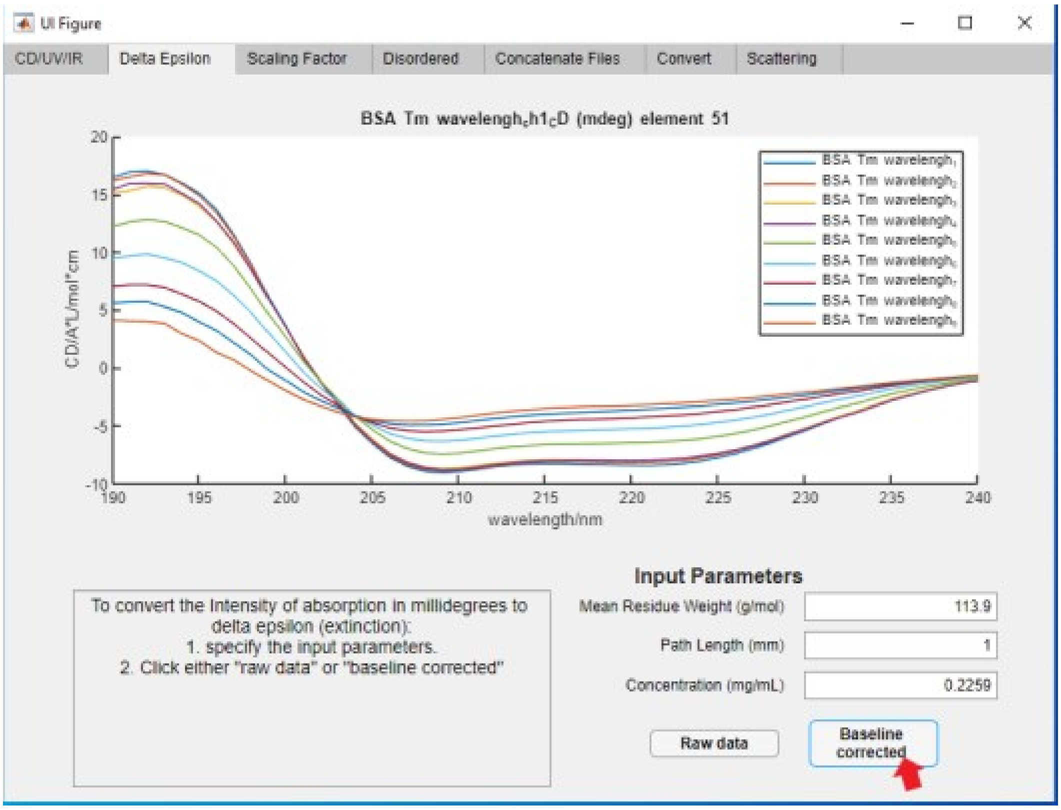

9) Switch to the **Disordered** module to take out a different percentage of the random coil from the other structures. Open the delta epsilon output file named "BCDEBSA Tm wavelength scans_ch1_CD(mdeg)," and insert the random coil spectrum into column two to make the spectra in this file ten. Save this modified file with an appropriate name (e.g. BCDEBSARC T wavelength scans_ch1_CD(mdeg)) in the New folder. Click the "select" push button to choose the saved file. Then type the fraction of disordered in the edit numeric box, as shown in the screenshot. After that, hit the "plot" button to generate the corresponding plot, xls, and txt files with name ending with decimal fraction expressed using a standard form (e.g. 1e-01), denoting the fraction of disordered removed. Repeat these steps for other fractions of disordered e.g. between 0.2–0.9 in 0.1 increments using the same input file. By simply typing the next fraction in the edit numeric box and hit plot button again

Please do not adjust margins

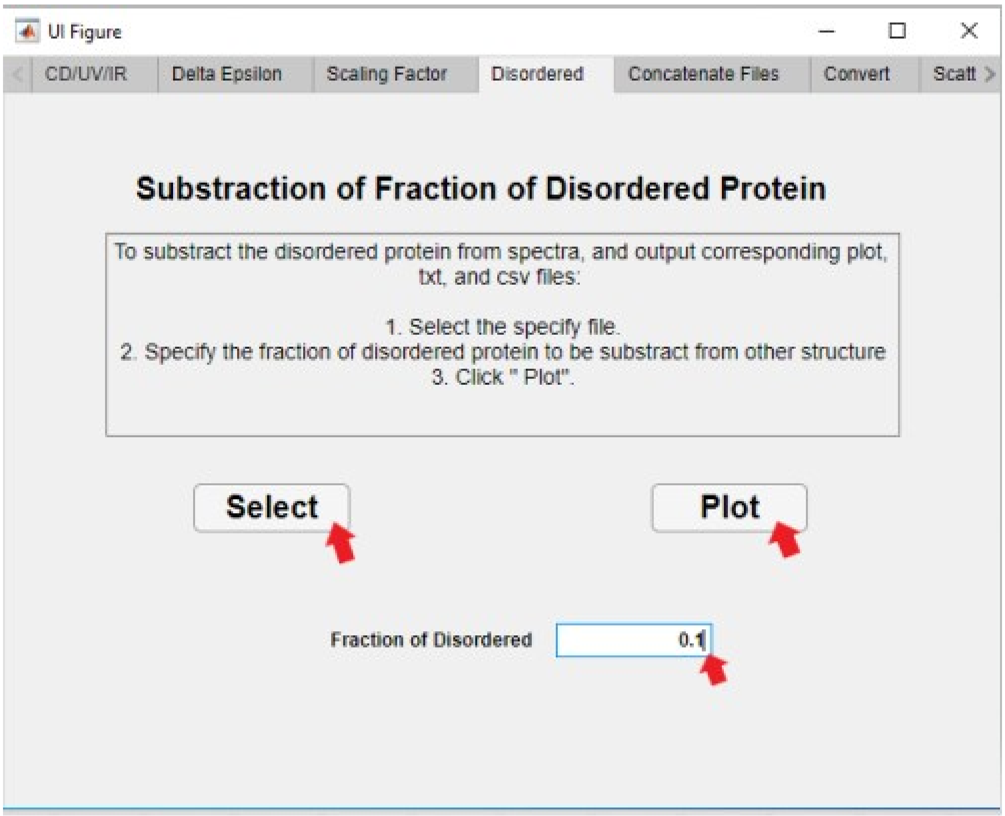

10) Switch to the **Concatenate** module to merge the xls output files of the disordered module in sequential order from the least to most percentage random coil, without including the wavelength column data for each file in the output txt file. For this work, first hit on “select file 1” push button to pick file with a name ending with 1e-01, then the second push button to pick file with a name ending with 2e-01, and third push button to pick file with a name ending with 3e-01. After these, click on the concatenate button to merge these files into a single xls and txt files named concatenate. Rename these two files by add 1 to the end of its name to indicate the first merged files. Repeat the above steps for the remaining files and add 2, and 3 to the names of subsequently merged files, and then combine the three output xls files with name ending with 1,2 and 3 into a single file containing 81 spectra. Rename this final file BSA Tm wavelength (0.1–0.9).

 This journal is © The Royal Society of Chemistry 20xx

Please do not adjust margins

Please do not adjust margins

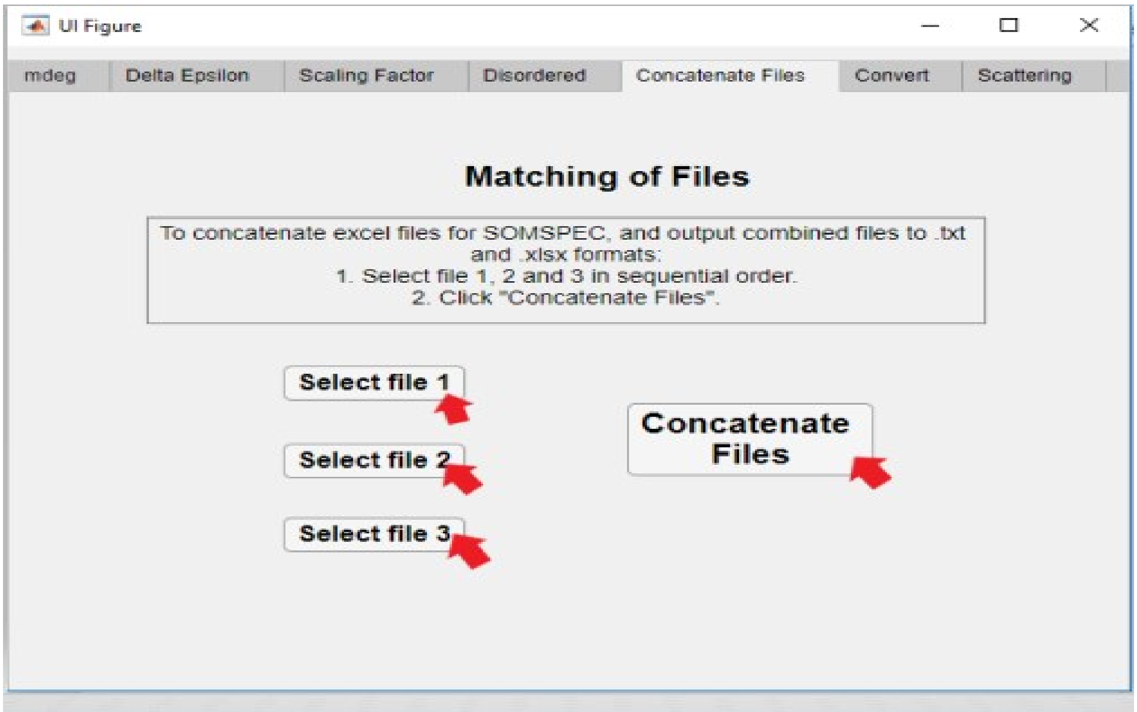

11) Ensure the input files for SOMSPEC are in txt format, and meant to be without column labels and wavelengths' data. Please note these files must have wavelength ranges corresponding to that of the reference set to be used. The step size does not matter.

12) Launch SOMSPEC and click on the **Train** module to train it with a reference set of secondary structures. Select the training set file named "SP175_full_240-190_5Pplus random a& 100 helix2" in txt format by clicking the "select training set file" push button. Then specify the input parameters: for this work, map size(50x50), number of iteration (50,000), number of structures (5), number of best matching units (5), wavelength range (240–190 nm). Check that the numbers on the right-hand side of the window mirror the input numbers. Afterward, hit the Train SOM button to train with the self-organizing map, and this output a pretrained SOM folder. The Module will output a folder named <SOM-50-50,000>. You can rename it to indicate the identity of the reference set used to train it.

13) Once you have a trained map you can use it again by entering the input parameters in the Train tab as above before moving to the Predict module (tab) where you select your previously trained Map..

14) Switch to the Predict module and click the select button to choose the pretrained SOM folder (SOM-50-50,000) containing trained maps, set parameters, and training set size information. Click on the "select input spectra" button to choose the data file named, e.g., "BSA Tm wavelength (0.1-0.9)," and check the <disable scaling of spectra> box. Then, hit the run prediction button to predict spectra and secondary

Please do not adjust margins

structures using the pretrained SOM, and this output predicted spectra, secondary structures, NRMSD, and other files.

15) Return to the CDspectra app and switch to the **Convert** module. The outputted real and predicted spectra of the SOMSPEC are without wavelength data. For the current example, they are arranged as 81 x 200 matrices (the rows correspond to spectra with 200 data points). This module transposes these files to 200 x 81 matrices, and it includes a column for wavelengths' data. Each column from the second column represents the BSA spectrum at a specific temperature and percentage random coil. Click on "select spectra_ real" to choose this file, and on "select spectra_predicited" to choose the predicted spectra. Then, hit on the convert button to transpose these data to vertical vectors and generate a linearly spaced vector of wavelengths between 240- 190 nm with 200 elements. It outputs xls files are named "TSpectra_real" and "TSpectra_predicited."

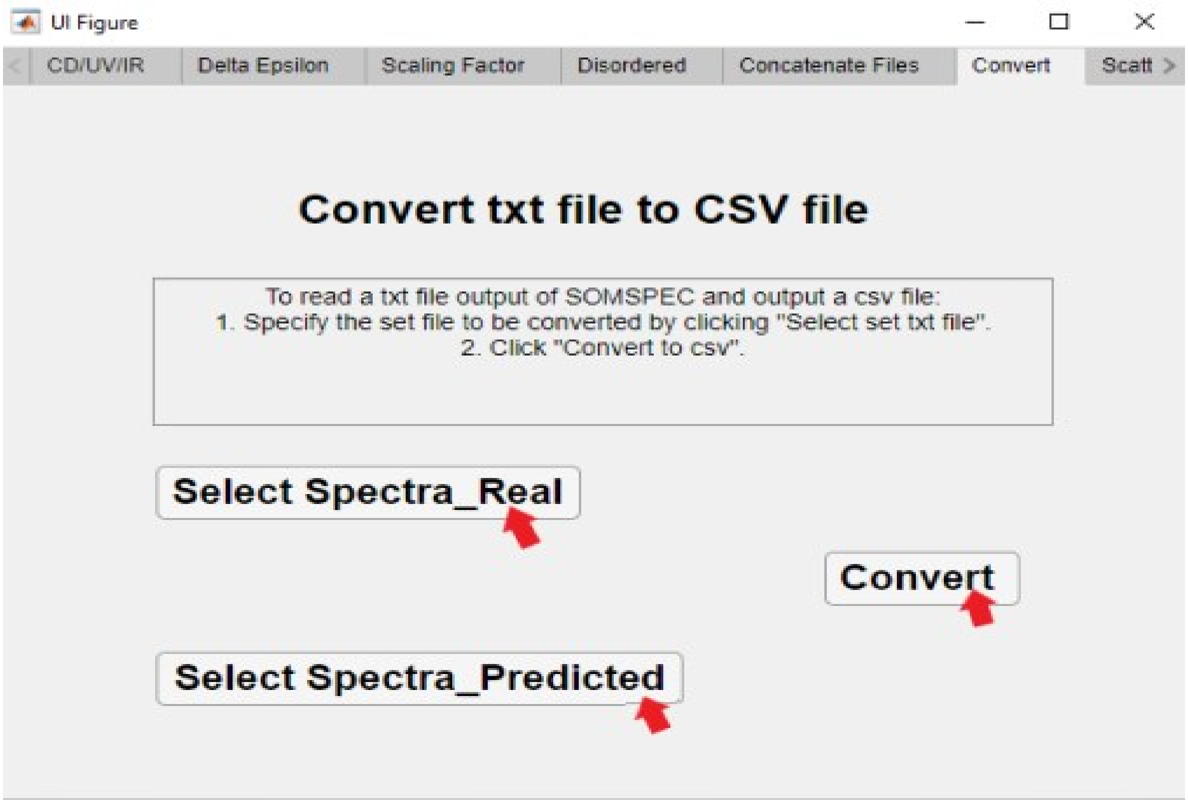

16) Repeat steps 1–15 for any other data set, adapting the instructions if the experimental data presentation is different. If the random coil adjustment is not required, then the data step of the spectra data does not matter, though the data range of the test spectra and the reference set must match.

 This journal is © The Royal Society of Chemistry 20xx

Please do not adjust margins

MATLAB CDSpectra Code

CD/UV/IR: code for baseline correction of spectra.

```
% Button pushed function: SelectSetfileButton
function SelectSetfileButtonPushed(app, event)

[file, path] = uigetfile('*.xlsx');
[filepath, filename, fileext] = fileparts(file);
app.filename = filename;
[app.num app.txt] = xlsread(app.filename)
end
% Value changed function: SpectrocopyListBox
function SpectrocopyListBoxValueChanged(app, event)
%value = app.SpectrocopyListBox.Value;
switch app.SpectrocopyListBox.Value
        case 'CD'
app.plot
title(app.UIAxes, app.filename);
legend(app.UIAxes,app.txt1);
xlabel (app.UIAxes,'wavelength/nm'); ylabel (app.UIAxes,'CD/mdeg');
        case 'UV'
app.plot
title(app.UIAxes, app.filename);
legend(app.UIAxes,app.txt1);
xlabel (app.UIAxes,'wavelength/nm'); ylabel (app.UIAxes,'Absorbance');
        case 'IR'
app.plot
title(app.UIAxes, app.filename);
legend(app.UIAxes,app.txt1);
xlabel (app.UIAxes,'wavenumber/cm-1'); ylabel
(app.UIAxes,'%Transmittance')
end
% Button pushed function: BaselinecorrectedButton
function BaselinecorrectedButtonPushed(app, event)
app.txt1 = app.txt(:,3:end);
app.numbc = app.num(:,3:end)- app.num(:,2);
app.plot2 = plot(app.UIAxes, app.num(:,1),app.numbc);
%title(app.UIAxes,app.filename);
```

This journal is © The Royal Society of Chemistry 20xx

Please do not adjust margins

Please do not adjust margins


```
fileName = strcat('BCMD',app.filename);
N = [app.txt(:,1),app.txt1];
data_cellsbc = num2cell(app.numbc);data_cellsn = num2cell(app.num(:,1));
M = [data_cellsn,data_cellsbc];
J = [N;M];
xlswrite(fileName,J);
end
end
```

Delta Epsilon: code for conversion of CD spectra in units mdeg to delta epsilon.

```
% Button pushed function: RawdataButton_2

function RawdataButton_2Pushed(app, event)

c = app.PathLengthmmEditField.Value;

d = app.ConcentrationmgmLEditField.Value;

M = app.MeanResidueWeightgmolEditField.Value;

data = app.num(:,3:end)*M/(32980*c*0.1*d);

plot(app.UIAxes2,app.num(:,1),data);

title(app.UIAxes2,app.filename);

legend(app.UIAxes2,app.txt1);

xlabel (app.UIAxes2,'wavelength/nm'); ylabel (app.UIAxes2,'CD/A*L/mol*cm');

fileName = strcat('RDDE',app.filename);

data_cellsrd = num2cell(data);data_cellsn= num2cell(app.num(:,1));

N = [app.txt(:,1),app.txt1];

M = [data_cellsn,data_cellsrd];

J = [N;M];

xlswrite(fileName,J);

end


% Value changed function: MeanResidueWeightgmolEditField

function MeanResidueWeightgmolEditFieldValueChanged(app, event)

value = app.MeanResidueWeightgmolEditField.Value;

end

% Value changed function: PathLengthmmEditField
```


This journal is © The Royal Society of Chemistry 20xx

Please do not adjust margins

Please do not adjust margins


```
function PathLengthmmEditFieldValueChanged(app, event)
value = app.PathLengthmmEditField.Value;
end

% Value changed function: ConcentrationmgmLEditField
function ConcentrationmgmLEditFieldValueChanged(app, event)
value = app.ConcentrationmgmLEditField.Value;
end

% Button pushed function: BaselinecorrectedButton_2
function BaselinecorrectedButton_2Pushed(app, event)
c = app.PathLengthmmEditField.Value;
d = app.ConcentrationmgmLEditField.Value;
M = app.MeanResidueWeightgmolEditField.Value;
data1 = app.numbc*M/(32980*c*0.1*d);
plot(app.UIAxes2,app.num(:,1),data1);
title(app.UIAxes2,app.filename);
legend(app.UIAxes2,app.txt1);
xlabel (app.UIAxes2,'wavelength/nm'); ylabel (app.UIAxes2,'CD/A*L/mol*cm');
fileName = strcat('BCDE',app.filename);
N = [app.txt(:,1),app.txt1];
data_cellsbc = num2cell(data1);data_cellsn = num2cell(app.num(:,1));
M = [data_cellsn,data_cellsbc];
J = [N;M];
xlswrite(fileName,J);
end
```

This journal is © The Royal Society of Chemistry 20xx

Please do not adjust margins

Please do not adjust margins

Scaling Factor: code for scaling of CD spectra using given factor(s) for concentration or conversion to delta epsilon.

```
% Button pushed function: SelectfileButton

function SelectfileButtonPushed(app, event)

[file, path] = uigetfile('*.xlsx');

[filepath, filename, fileext] = fileparts(file);

app.fileName = filename

[num txt raw] = xlsread(app.fileName)

app.scale1 = num(:,1);

app.scale2 = num(:,2:end);

app.txts1 = txt(:,1);

app.txt2 = txt(:,2:end);

end

% Value changed function: ScalingfactorEditField

function ScalingfactorEditFieldValueChanged(app, event)

app.scalingfactor = app.ScalingfactorEditField.Value;

end
% Button pushed function: PrintButton

function PrintButtonPushed(app, event)

hFig = figure;
ax = axes('Parent', hFig);

app.ScalefileName4 = app.scale2.*app.scalingfactor;

S = sprintf('%.d', app.scalingfactor)

 %S = num2str(app.scalingfactor);

 filename = strcat(app.fileName, S);

 plot(ax,app.scale1,app.ScalefileName4);

 xlabel(ax,'wavelength/nm');ylabel(ax,'M-1cm-1');

 title(ax, filename);

 legend(ax, app.txt2);

 saveas(hFig, filename,'pdf');
```

 This journal is © The Royal Society of Chemistry 20xx

Please do not adjust margins

Please do not adjust margins


```
 num1 = num2cell(app.scale1); num2 = num2cell(app.ScalefileName4);
 H =[app.txts1,app.txt2;num1, num2]
 xlswrite(filename,H);
 end
```

Disordered: code for derandomization of spectra by removing varying fractions of random coil spectrum.

```
% Button pushed function: SelectButton
function SelectButtonPushed(app, event)
[file, path] = uigetfile('*.xlsx');
[filepath, filename, fileext] = fileparts(file);
app.filenamess = filename;
[num txt raw]  = xlsread(app.filenamess);
app.wavelength = num(:,1)
app.dataR  = num(:,2)
app.dataS = num(:,3:end)
app.txtN1 = txt(:,1)
app.txtD = txt(:,3: end)
end
% Value changed function: FractionofDisorderedEditField
function FractionofDisorderedEditFieldValueChanged(app, event)
app.fractiondisordered = app.FractionofDisorderedEditField.Value;
end
% Button pushed function: PlotButton_2
function PlotButton_2Pushed(app, event)
hFig = figure; ax = axes('Parent',hFig);
DataSD = (app.dataS - app.fractiondisordered*app.dataR)/(1-app.fractiondisordered)
S = sprintf('%.d', app.fractiondisordered)
filename = strcat(app.filenamess,S);
plot(ax, app.wavelength, DataSD);
xlabel(ax,'wavelength/nm'); ylabel(ax,'M-1cm-1');
```


Please do not adjust margins

Please do not adjust margins


```
title (ax,app.filenamess);

legend(ax,app.txtD);

saveas(hFig,filename,'pdf');

Wavelength = num2cell(app.wavelength)

Data_SD = num2cell(DataSD)

app.txtN1

app.txtD

L = [app.txtN1,app.txtD; Wavelength, Data_SD]

M = [app.wavelength, DataSD];

xlswrite(filename,L);

dlmwrite(filename,M);

end
```

Concatenate: code for matching xlsx files together

```
% Button pushed function: Selectfile1Button

function Selectfile1ButtonPushed(app, event)

[file, path] = uigetfile('*.xlsx');

[filepath, filename, fileext] = fileparts(file);

[app.num4 txt  raw] = xlsread(filename);app.raw = raw;

End

% Button pushed function: Selectfile2Button
function Selectfile2ButtonPushed(app, event)

[file, path] = uigetfile('*.xlsx');

[filepath, filename, fileext] = fileparts(file);

[app.num5 txt  raw1] = xlsread(filename); app.raw1 = raw1;

end

% Button pushed function: Selectfile3Button
function Selectfile3ButtonPushed(app, event)

[file, path] = uigetfile('*.xlsx');

[filepath, filename, fileext] = fileparts(file);

[app.num6 txt  raw2] = xlsread(filename);app.raw2 = raw2;
```


This journal is © The Royal Society of Chemistry 20xx

Please do not adjust margins

Please do not adjust margins


```
end

% Button pushed function: ConcatenateFilesButton
function ConcatenateFilesButtonPushed(app, event)

J = [app.num4(:,2:end),app.num5(:,2:end),app.num6(:,2:end)];

H = [app.raw,app.raw1(:,2:end),app.raw2(:,2:end)];

dlmwrite('concatenate file.txt',J);

xlswrite('concatenate file.xlsx',H);

end
```

Convert: code for the reading of txt files output of SOMSpec and output xlsx files

```
% Button pushed function: SelectSpectra_RealButton

function SelectSpectra_RealButtonPushed(app, event)

fileN = uigetfile('*.txt');

app.Select1 = dlmread(fileN);

end

% Button pushed function: SelectSpectra_PredictedButton

function SelectSpectra_PredictedButtonPushed(app, event)

fileN1 = uigetfile('*.txt');

app.Select2 = dlmread(fileN1);

end

% Button pushed function: ConvertButton
function ConvertButtonPushed(app, event)

fileName = strcat('T', 'Spectra_Real');

fileNameI = strcat('T', 'Spectra_Predicted');

Spectra_Real = app.Select1';

Spectra_Predicted = app.Select2';

T = linspace(240,190,200);

Wavelength = T';

H = [Wavelength, Spectra_Real]

I = [Wavelength, Spectra_Predicted]

xlswrite(fileName,H);

xlswrite(fileNameI,I);

end
```


Please do not adjust margins

Please do not adjust margins

Notes and references

1 Whitmore, L. & Wallace, B. A. DICHROWEB: an online server for protein secondary structure analyses from circular dichroism specroscopic data. *Nuc. Acids Res.* **32**, W668-673 (2004).

This journal is © The Royal Society of Chemistry 20xx

Please do not adjust margins
